# Investigation of Event-Based Surfaces for High-Speed Detection, Unsupervised Feature Extraction, and Object Recognition

**DOI:** 10.3389/fnins.2018.01047

**Published:** 2019-01-17

**Authors:** Saeed Afshar, Tara Julia Hamilton, Jonathan Tapson, André van Schaik, Gregory Cohen

**Affiliations:** Biomedical Engineering and Neuroscience Program, The MARCS Institute for Brain, Behaviour, and Development, Western Sydney University, Sydney, NSW, Australia

**Keywords:** event-based vision, recognition and classification, neuromorphic, event-based, unsupervided learning

## Abstract

In this work, we investigate event-based feature extraction through a rigorous framework of testing. We test a hardware efficient variant of Spike Timing Dependent Plasticity (STDP) on a range of spatio-temporal kernels with different surface decaying methods, decay functions, receptive field sizes, feature numbers, and back end classifiers. This detailed investigation can provide helpful insights and rules of thumb for performance vs. complexity trade-offs in more generalized networks, especially in the context of hardware implementation, where design choices can incur significant resource costs. The investigation is performed using a new dataset consisting of model airplanes being dropped free-hand close to the sensor. The target objects exhibit a wide range of relative orientations and velocities. This range of target velocities, analyzed in multiple configurations, allows a rigorous comparison of time-based decaying surfaces (time surfaces) vs. event index-based decaying surface (index surfaces), which are used to perform unsupervised feature extraction, followed by target detection and recognition. We examine each processing stage by comparison to the use of raw events, as well as a range of alternative layer structures, and the use of random features. By comparing results from a linear classifier and an ELM classifier, we evaluate how each element of the system affects accuracy. To generate time and index surfaces, the most commonly used kernels, namely event binning kernels, linearly, and exponentially decaying kernels, are investigated. Index surfaces were found to outperform time surfaces in recognition when invariance to target velocity was made a requirement. In the investigation of network structure, larger networks of neurons with large receptive field sizes were found to perform best. We find that a small number of event-based feature extractors can project the complex spatio-temporal event patterns of the dataset to an almost linearly separable representation in feature space, with best performing linear classifier achieving 98.75% recognition accuracy, using only 25 feature extracting neurons.

## Introduction

The last decade has seen significant development in the field of event-based cameras. Cameras such as the Dynamic Vision Sensor (DVS) (Lichtsteiner et al., 2008) and the Asynchronous Time-based Image Sensor (ATIS) (Posch et al., 2011) attempt to model the operation of the human retina by generating events at each pixel in response to changes in illumination. By only reporting changes in the visual field, event-based sensors perform compressive sensing at the pixel level, significantly reducing the output data-rate of the sensor relative to frame-based sensors that generate output regardless of the salience of its visual content. These cameras have spurred the development of a range of visual processing algorithms to tackle existing problems such as optical flow detection (Benosman et al., 2012), scene stitching (Klein et al., 2015), motion analysis (Litzenberger and Sabo, 2012), hand gesture recognition (Lee et al., 2014), hierarchical feature recognition (Orchard et al., 2015b), unsupervised visual feature extraction, and learning (Giulioni et al., 2015; Lagorce et al., 2015a), and tracking (Lagorce et al., 2015b; Glover and Bartolozzi, 2016, 2017). In addition to these works, in Ghosh et al. (2014) a frame based convolutional neural network was mapped to an event-based network using conversion of the event stream to static images via recent event presence, event counts, and event polarity. In Zhao et al. (2015), a hierarchical feature extractor network is presented where manually designed features are based on models of features in the visual cortex. In Peng et al. (2017), a bag of events method is used to perform feature extraction. An especially useful feature of this method is that only a single hyper-parameter needs to be tuned. This is in contrast to most proposed methods, which often have a large number of parameters, such that a rigorous analysis of their performance requires careful characterization and/or adversarial parameter selection, both of which are performed in this work.

More recently, the Hierarchy of Time Surfaces (HOTS) (Lagorce et al., 2017) was introduced which makes use of layers of time-decaying event-surfaces, or time surfaces, and feature-based clustering, with the features learnt in an unsupervised manner. The HOTS approach processes events in the temporal domain and is functionally similar to the feature extraction layer used in this work. The time surfaces which are used in HOTS and which also form part of the investigation in this work are a particularly effective method of implementing event-based convolutional networks.

In this work, we set out to rigorously quantify in detail the share in performance improvement attributable to each element of the system, namely: the memory generation and decay methods, commonly used memory kernels, use of raw events relative to use of feature events, the event-based convolutional structure of the feature extractors and the performance of the back-end classifier.

An important question arising at every stage of any event-based algorithm is whether the event rate should inform the progression of the algorithm through time. In this work, we investigate this question through comparisons of time surfaces and index surfaces where the memory of events decay as a function of time or event index, respectively.

Processing event memory as a function of time is straight-forward and intuitive. By decaying event memory as a function of time, all elements of an event-based system operate in a uniform time-based manner regardless of the informational content in any part of the sensor's field of view. The behavior of time-based decaying memory does not vary as a function of sensor size or any aspect of the visual scene that alters the event generation rate, such as scene contrast or texture. However, once the sensor event rate is incorporated into the operation of the system, these invariances may no longer hold, since a change in event rate may alter the decay rate of the memory of the event stream, potentially resulting in information loss. Therefore, algorithms using event rate information in memory decay require more careful testing, parameter selection, and potentially secondary solutions such as localized memory decay mechanisms to mitigate information loss. On the other hand, processing event memory as a function event count or index does have one crucial advantage over a purely time-based processing system. In general, event-based vision sensors generate more events in response to faster moving objects when holding other variables constant. This approximately proportional relationship between local event rate and local velocity allows an algorithm operating as a function of event index to effectively make computational decisions at approximately the same speed as the object being observed. Previous works have suggested that the use of event index to decay memory provides greater robustness in the presence of such variance in target velocity (Ghosh et al., 2014; Glover and Bartolozzi, 2016, 2017). In Glover and Bartolozzi (2016) an event-based Hough transform was used for tracking and in Ghosh et al. (2014) this was augmented with an event-based particle filter to improve tracking performance. The Hough transform in these works was implemented using a window of fixed event size, thus incorporating the event-rate information into the algorithm. The results showed that higher target velocities increased the update rate of the algorithm, allowing better tracking performance at high velocity. In Ghosh et al. (2014), windows of fixed event number and fixed time windows were compared in their performance in simultaneous tracking and recognition, and a slightly higher recognition accuracy was achieved when the algorithm was tested for velocity invariance. Such robustness to observed velocities in the data can be critical in a range of real world applications. These results, and the potential utility of velocity robust algorithms in real world applications of event-based sensors, motivate a central element of the investigation presented in this work. One such example is one of the few current applications of event-based sensors: the field of event-based Space Situational Awareness (SSA), where event-based sensors uniquely allow observation and tracking of non-terrestrial targets during both night and day (Cohen et al., 2017). However, a major challenge in such a task is the extremely limited collection of event-based observations of objects of interest. A major aspect of this limitation is that particular targets may only have been observed at a single velocity relative to the sensor yet must be detected, tracked, and identified robustly regardless of their relative velocity. This requirement of robustness to target velocity variations motivates the detailed rigorous examination of time and index surfaces in combination with a range of commonly used decay kernels.

Another important element in a wide range of event-based algorithms is the use of feature extractors. The contribution of the feature extraction layer as a whole is the simplest to determine and yet can often be missing in the literature as a baseline performance measure. This involves directly feeding sensor events into the final stage classifiers in the same manner as the output feature layer, skipping the intervening feature extraction layer(s). A more subtle question is how effective the learnt features are. In other words, how well does the learning algorithm orient the feature set with respect to the data so as to cover the underlying non-linearities in the dataset? This can be ascertained by comparing the mean recognition performance of multiple independently learnt features against random instantiations of features with the same network structure and feature weight distribution. The power of random features to cover non-linear feature spaces has been demonstrated by the Extreme Learning Machine (Clady et al., 2015) literature. By comparing feature extraction algorithms to a baseline of random features a better understanding of the relative improvement can be ascertained.

Finally, the most complex measure that is investigated is the role of the classifier on the performance. While there are a wide range of potential back-end classifiers that may be used, we propose that the combined use of linear classifiers and large hidden layer ELMs have particular utility in providing a rigorous measure of residual non-linearity following each stage of processing. This is because, unlike other classifiers, which through learning orient their non-linear features toward the training data, the random non-linear projections of the ELM's hidden layer create projections that are approximately uniform with regard to the structure of the data. As such the size of the hidden layer provides a reasonably “unbiased” measure of the residual non-linearities present after each a processing layer.

## Methodology

### Generating the Dataset

The system presented in this paper constitutes an event-based and high-speed classification system, and makes use of a real-world task, and its associated dataset, to demonstrate and characterize its performance.

A variety of event-based datasets now exist, such as the N-MNIST and N-Caltech101 (Orchard et al., 2015a), MNIST_DVS (Serrano-Gotarredona and Linares-Barranco, 2015), and the event-based UCF-50 datasets (Hu et al., 2016). One common facet of these datasets is that they have been generated under highly constrained conditions, especially with respect to the range of target object velocities. For a static image, event-based cameras only produce data in response to motion and therefore require either the static image, or the camera itself to be moving. Therefore, the velocities involved in many of the event-based datasets are strictly controlled. This is often a desirable trait to ensure consistency across all samples, but this constraint is a strongly artificial one. Other event-based datasets, such as the visual navigation dataset found in Barranco et al. (2016), do not control velocity in the same manner, but represent a fundamentally different task and are therefore not well-suited to exploring detection and feature extraction mechanisms.

The need to explore the effect of variances in velocity is important as these tend to produce significant variance in the spatio-temporal event patterns generated by event-based cameras. This can have a significant impact on the performance of a classifier or detection algorithm. A primary focus of this work is on the comparison of different event-based processing approaches in the presence of such variance. This required the creation of a new dataset designed to test event-based classification algorithms under conditions that are less constrained and closer to those found in real-world tasks. However, as well as being reasonably difficult, the dataset was also designed to be constrained enough to allow a rigorous comparison of the various parameters and architectures of interest. As such the dataset was specifically designed to act as a proxy for a noisy local region in a larger real-world dataset.

**Figure 1:**
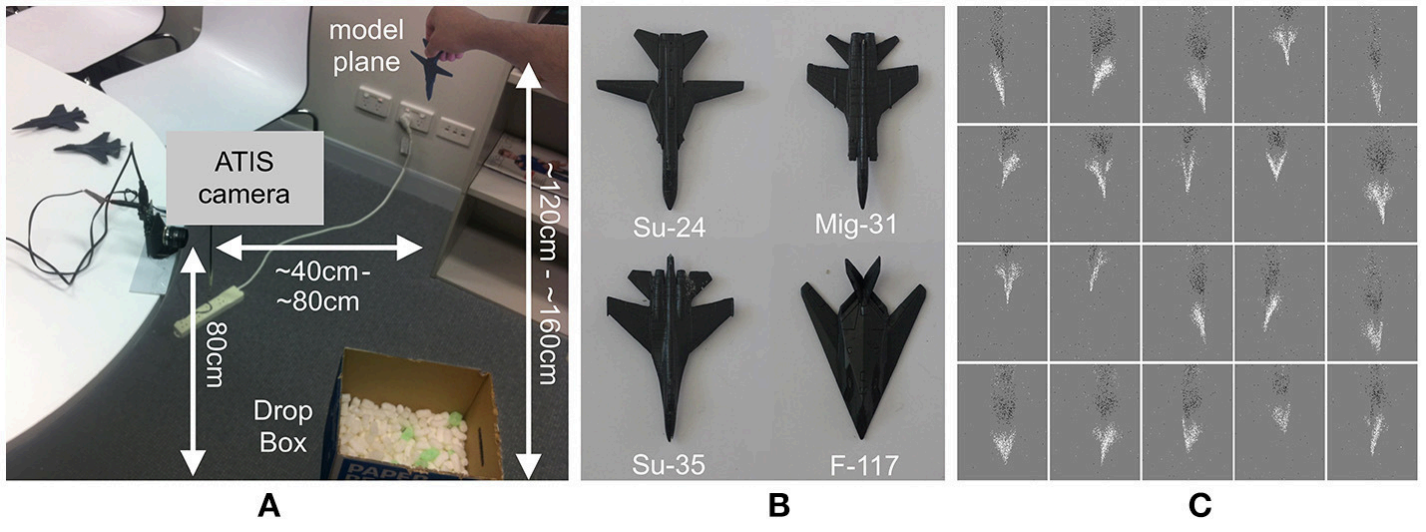
Data collection setup and samples of the airplane dropping dataset. **(A)** The physical setup used for recording dataset in which an ATIS camera is attached to a table and the airplanes dropped freehand in front of the camera. **(B)** A top-down and labeled view of the four model airplanes used to generate the dataset. **(C)** Examples of the variation in the dataset in terms of position, scale, orientation, and speed. Each image represents a frame rendered from the same 3 ms of events extracted from each recording with ON events represented with white pixels and OFF events represented with black pixels. The twenty random samples clearly demonstrate the difficulty of the recognition task. Unlike most event-based datasets, the camera was not tuned or biased for the application, simulating real world noisy dynamic environments where such fine tuning would be difficult or impossible. As a result of this arbitrary untuned camera configuration the OFF events (black) in the entire dataset produced essentially noise clouds and as such were discarded. Airplane class key ordered from top left to bottom right, Mig-31: {2, 3, 7, 11, 12}, F-117: {9, 15, 16, 18, 19}, Su-24: {1, 5, 8, 14, 20}, and Su-35: {4, 6, 10, 13, 17}.

The task is to identify model airplanes as they rapidly pass through the field of view of an ATIS camera. The airplanes were dropped free-hand, and from varying heights and distances from the camera, as shown in Figure 1A. Four model airplanes were used, each made from steel and all painted uniform gray, as shown in Figure 1B. This served to remove any distinctive textures or marking from the airplanes, thereby increasing the difficulty of the task. The airplanes are models of a Mig-31, an F-117, a Su-24, and a Su-35, with wingspans of 9.1, 7.5, 10.3, and 9.0 cm, respectively.

The recordings were captured using the same model of ATIS camera and the same acquisition software used in capturing the N-MNIST dataset in Orchard et al. (2015a), and the recordings were stored in the same file formats, thereby maximizing compatibility with other neuromorphic algorithms and systems. The models were dropped 100 times each from a distance ranging from 120 to 160 cm above the ground and at a horizontal distance of 40 to 80 cm from the camera. This ensured that the airplanes passed rapidly through the field of view of the camera, with the planes crossing the field of view in an average of 242 ± 21 ms. No mechanisms were used to enforce consistency of the airplane drops, resulting in a wide range of observed speeds from 0 to >1500 pixels per second. Additionally, there were variable delays before and after each drop, resulting in recordings of varying lengths. The dataset was augmented with left-right flipped versions of the recordings, resulting in 200 drops for each airplane type. An example of the variability in the airplane drops is demonstrated in Figure 1C, which shows binned events in the same 3 ms slice of data from 20 randomly selected recordings from the dataset. The samples demonstrate significant variations in the positions of the airplanes, their orientations, and their sizes. No attempt was made to fine tune the sensors biases for the particular light condition or target velocities. This lack of tuning is likely in real-world environments where the recording conditions may not be known a priori. An example of this is the previously mentioned SSA application (Cohen et al., 2017), where acquired data is inevitably noisy, often with one of sensors polarities entirely unable to capture useful events from the target due to the sensor biases not being matched to the lighting or velocity profile of the target. Even when the sensor biases are ideal for the lighting and temperature conditions of the recording, there are always fainter targets of interest in the field of view which can only be viewed by lowering sensor biases and “delving deeper into the noise” to accumulate events from these fainter objects. Thus, allowing noise and un-tuned biases into datasets, additional real-world challenges, such as structured noise and unevenly performing polarities, become apparent, motivating the implementation of robust solutions and new network behaviors that would otherwise be missed.

**Figure 2:**
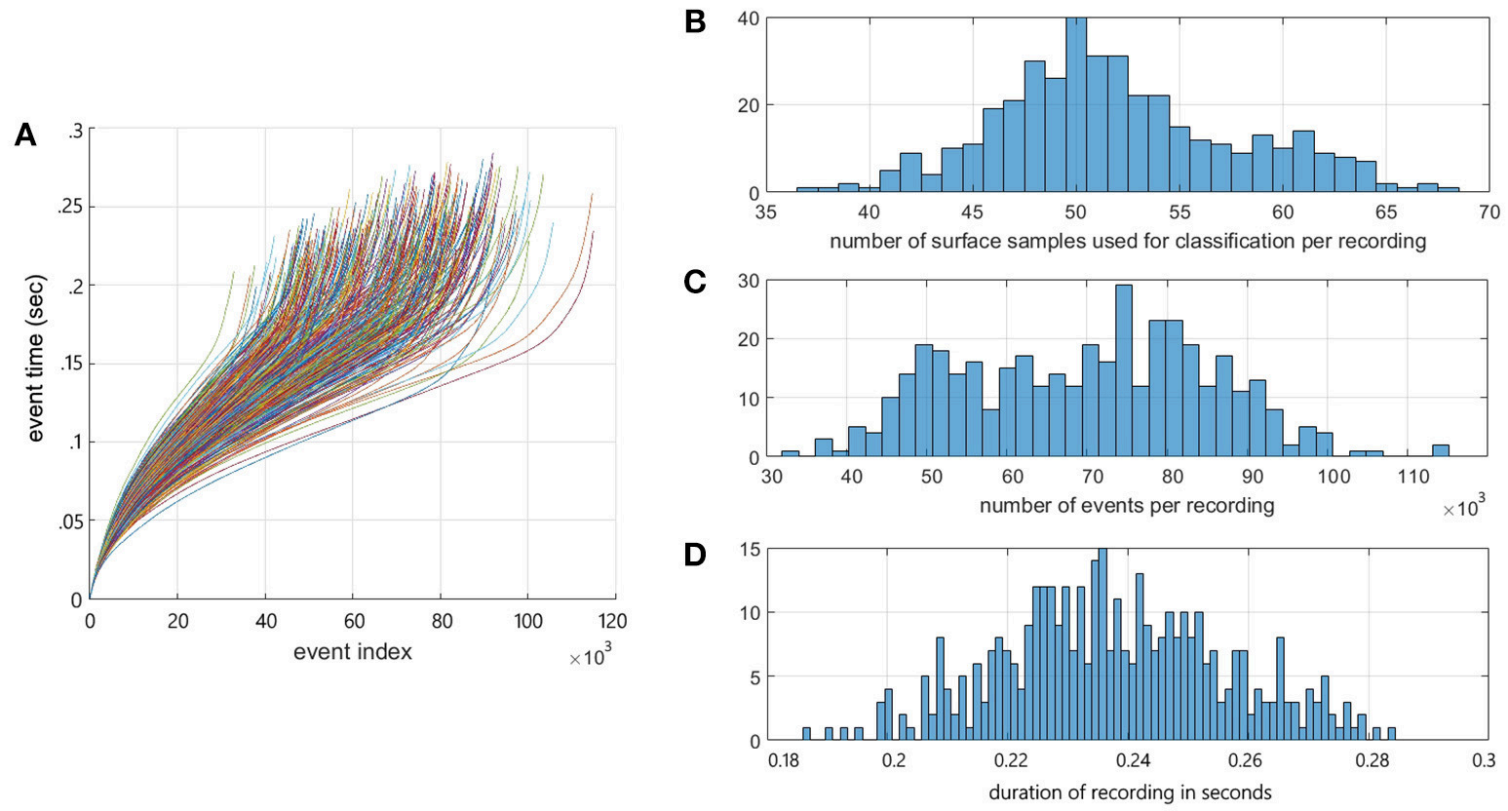
The Dataset Summary. **(A)** Event timestamp profiles of all airplane drops in the dataset showing the event timestamps of each recording as a function of event index. The timestamp profiles demonstrate the variable rates of event generation within and across the recordings. These differences are a function of the speed, size, and shape of the airplanes and the distance from the camera. Note the color assigned to each recording profile is arbitrary. **(B)** Distribution of the number of frames per recording for each recording in the dataset. **(C)** Distribution of the number of events per recording for each recording in the dataset. **(D)** Distribution of the duration of each recording in the dataset.

Figure 2A shows the event time vs. event index profiles of all recordings in the dataset showing the significant inter and intra recording variance in data-rate present in the dataset. While the number of recordings in the augmented dataset is 800, the number of surface samples making up the data points presented to the detection and recognition algorithm is >20,000 samples. The free-hand drop methodology resulted in significant variance in velocity and orientation of the model airplane within each recording. As a result, the spatio-temporal output patterns varied significantly through each recording, as shown in Figure 2A and discussed in later sections. The distribution of the number of surface samples per recording is shown in Figure 2B. Figures 2C,D show the distribution in the number of events per recording and recording duration for the dataset. The full dataset can be found at Afshar et al. (2018).

### Time-Surface vs. Index Surfaces

An event *ev*_*i*_ from the ATIS camera can be described mathematically by:

(1) $$\begin{array}{l} {ev_{i}=\left[{\text{x}}_{i},t_{i},p_{i}\right]}^{T} \end{array}$$

where *i* is the index of the event, **x**_**i**_ = [*x*_*i*_, *y*_*i*_] is the spatial address of the source pixel corresponding to the physical location on the sensor, *p*_*i*_ ∈ {−1, 1} is the polarity of event indicating whether the log intensity increased or decreased, and *t*_*i*_ is the absolute time at which the event occurred (Clady et al., 2015). The time *t*_*i*_ is applied to the event by the ATIS camera hardware and has a resolution of 1 ms.

Event-based algorithms require iterative processing of each event, and therefore require that each new observation be combined with previously observed local events, both in space and in time. This is accomplished using a variation of the time surfaces from the HOTS algorithm (Lagorce et al., 2017), but extended to cover surfaces decaying based on time (time surface) and based on event index (index surface). Each new incoming event updates the surface and defines a region representing the spatio-temporal neighborhood on which further processing may be performed.

The timing and polarity information contained in each event, as shown in equation (1), allows the generation of two useful surfaces, based on time and polarity, from which more complex surfaces can be constructed. The first surface, referred to as *T*_*i*_, maps the time of the most recent event to spatial pixel location and is described in (2), with the corresponding surface *P*_*i*_ for event polarity given by (3). Note as discussed above due to the noisiness of the OFF events due to untuned biases, only ON events with *p*_*i*_ = 1 were used.

(2) $$\begin{array}{l} T_{i}:{\text{R}}^{2}\to \text{R}\\ \text{x}\;:t\to \;T_{i}\left(\text{x}\right) \end{array}$$

(3) $$\begin{array}{l} P_{i}:{\text{R}}^{2}\to \left\{-1,1\right\}\\ \text{x}:p\to P_{i}\left(\text{x}\right) \end{array}$$

Here, we compare the time surfaces introduced in the HOTS algorithm, which decay as a function of time, with index surfaces, where the surface values for all pixels decay not as a function of time, but in response to new incoming events. We then define the analogous function to (2) for index surfaces. This surface, *I*_*i*_, is defined in (4) and stores the indices of incoming event for each spatial pixel.

(4) $$\begin{array}{l} I_{i}:{\text{R}}^{2}\to \text{R}\\ \text{x}:i\to \;I_{i}\left(\text{x}\right) \end{array}$$

In addition to exploring time-based decay and index-based decay, three different transfer functions or temporal kernels are investigated. These kernels are event binning (*BTS*/*BIS*), linear decay (*LTS*/*LIS*) and exponential decay (*ETS*/*EIS*). As a point of reference, the HOTS algorithm makes use of exponential decaying time kernels.

In all surface generation methods, when a new event arrives, the surface at **x**_**i**_ is set to *P*_*i*_. When using the event binning technique, the value on the surface maintains its value over a temporal window τ_*e*_ or index window *N*_*e*_, after which it is reset to zero. The event binning method for surface generation is described by equations (5) for the time-based binning (*BTS*) and (6) for the index-based binning (*BIS*).

(5) $$\begin{array}{lll} BTS_{i}\left(\text{x},t\right) & = & \{\begin{array}{c} P_{i}\left(\text{x}\right),\;{t-T}_{i}\left(\text{x}\right)\leq \tau_{e}\\ 0,\;t-\;T_{i}\left(\text{x}\right)>\tau_{e} \end{array} \end{array}$$

(6) $$\begin{array}{lll} BIS_{i}\left(\text{x}\right) & = & \{\begin{array}{c} P_{i}\left(\text{x}\right),\;{i-I}_{i}\left(\text{x}\right){\leq N}_{e}\\ 0,\;{i-I}_{i}\left(\text{x}\right){>N}_{e} \end{array} \end{array}$$

For the linearly decaying time surface (*LTS*) and linearly decaying index surface (*LIS*), the initial value set on the surface in response to a new event instead decays toward zero linearly as a function of time. These surfaces are described by (7) for time-based linear decay or in response to incoming events as described by (8) for index-based linear decay.

(7) $$\begin{array}{lll} LTS_{i}\left(\text{x},t\right) & = & \{\begin{array}{c} \begin{array}{cc} P_{i}\left(\text{x}\right).\left(1+\frac{T_{i}\left(\text{x}\right)-t}{2\tau_{e}}\right), & t{-T}_{i}\left(\text{x}\right)\geq {2\tau }_{e}\\ 0, & t{-T}_{i}\left(\text{x}\right)<{2\tau }_{e} \end{array} \end{array} \end{array}$$

(8) $$\begin{array}{lll} LIS_{i}\left(\text{x}\right) & = & \{\begin{array}{c} \begin{array}{cc} P_{i}\left(\text{x}\right).\left(1+\frac{I_{i}\left(\text{x}\right)-i}{2N_{e}}\right), & i-I_{i}\left(\text{x}\right)\geq {2N}_{e}\\ 0, & i-I_{i}\left(\text{x}\right)<{2N}_{e} \end{array} \end{array} \end{array}$$

The exponential decay method works in a similar manner to the linear decay, with the value placed on the surface decaying exponentially instead of linearly with respect to either time or event. This results in the equations for the exponentially decaying time surface (*ETS*) shown in (9), and the exponentially decaying index surface (*EIS*) shown in (10).

(9) $$\begin{array}{l} ETS_{i}\left(\text{x},t\right)=P_{i}\left(\text{x}\right).e^{\frac{T_{i}\left(\text{x}\right)-t}{\tau_{e}}} \end{array}$$

(10) $$\begin{array}{l} EIS_{i}\left(\text{x}\right)=P_{i}\left(\text{x}\right).e^{\frac{I_{i}\left(\text{x}\right)-i}{N_{e}}} \end{array}$$

**Figure 3:**
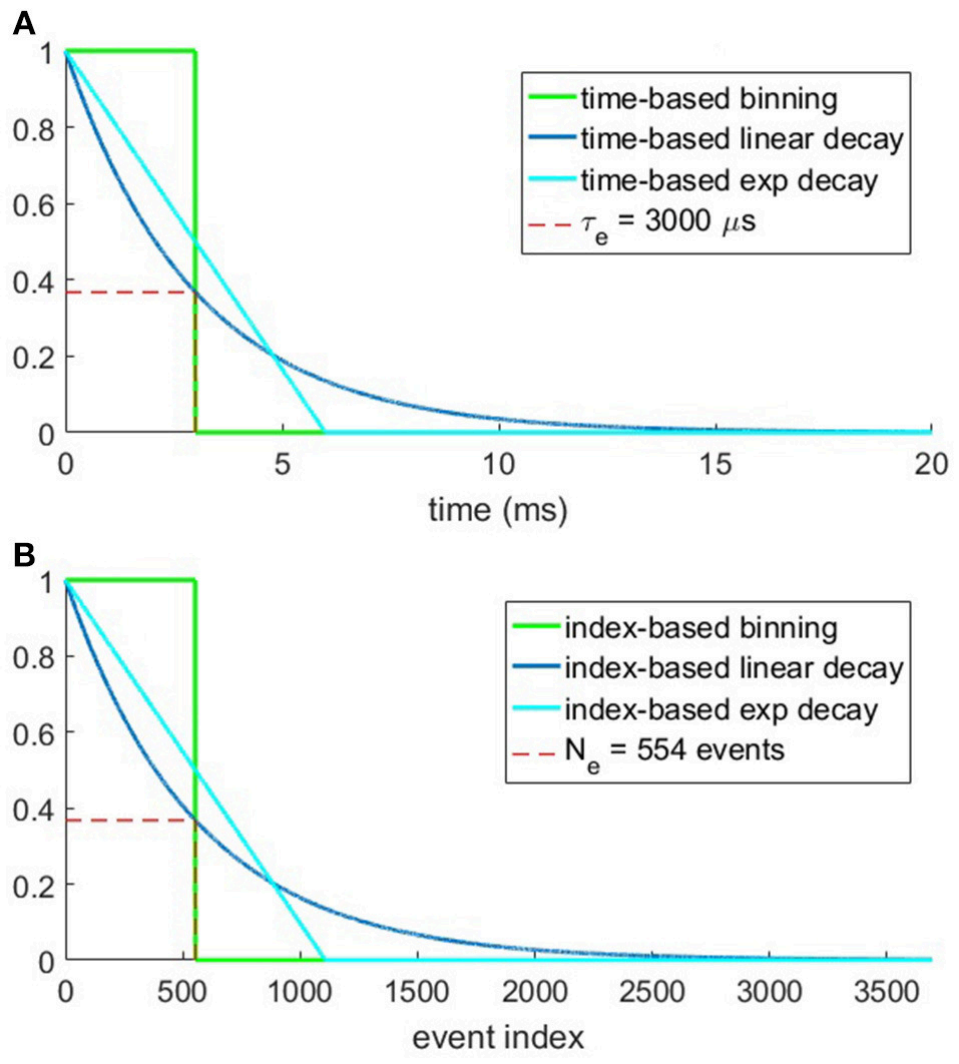
Plots of the six methods for generating time and index surfaces. **(A)** Shows the three time-based kernels over time. Note that the area under all kernels is the time constant τ_*e*_ = 3 ms. **(B)** shows the value of the index-based kernel as a function of event index. Here the mean dataset event rate over all recordings (~184.5 k events/s) was used to obtain equivalent sized kernels with index constant *N*_*e*_ = 554 events.

The equations for these surfaces make use of a constant parameter, time constant τ_*e*_ for time-based methods and index constant *N*_*e*_ for the index-based methods and the chosen values for these parameters are shown in Figures 3A,B. The plots show the time surface and index surface generation kernels which have an area under the curve of 3 ms in (a), and 554 events in (b), respectively. These values were chosen based on the mean data rate over all recordings.

Given the 184.5 k event/s event rate for the entire dataset the area under the curves in Figures 3A,B, τ_*e*_ = 3 and *N*_*e*_ = 554, respectively were chosen to be approximately equal, thus resulting in approximately equal total surface activation for the time and index based decay methods over the entire dataset, but not for any individual recording or section thereof.

**Figure 4:**
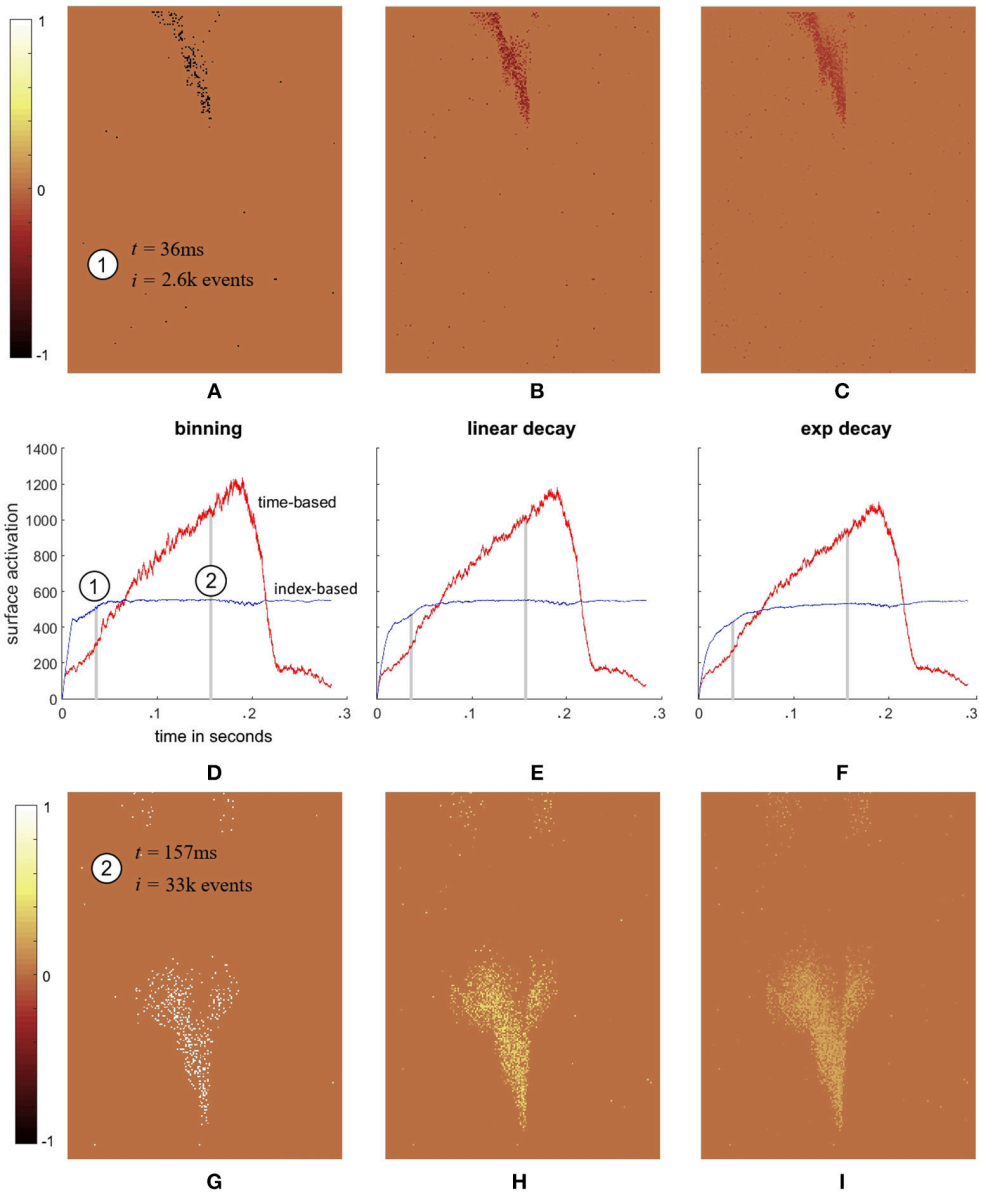
Comparison of surface activation for a single recording. **(A–C)** show the surface differences (*BTS*_*i*_ − *BIS*_*i*_), (*LTS*_*i*_ − *LIS*_*i*_), and (*ETS*_*i*_ − *EIS*_*i*_), respectively at the beginning of the recording (*t* = 36 ms). This moment in the recording is marked (1) on **(D)** which displays total surface activation for the binning method $\underset{x,y}{\sum }{BTS}_{i}$ and $\underset{x,y}{\sum }{BIS}_{i}$. The two traces in **(D)** show that at the beginning of the recording when the target airplane's speed is low the binning time surface has a lower activation than the binning index surface. However, as the target speeds up, the total time surface activation also increases, while the index surface remains approximately stable, such that by *t* = 157 ms the time surface activation $\underset{x,y}{\sum }{BTS}_{i}$ is approximately twice that of $\underset{x,y}{\sum }{BIS}_{i}$. **(E,F)** show a similar but slightly less pronounced relative increase for the linear and exponential decay surfaces. **(G–I)** show this relative increase for the binning, linear, and exponential decay surfaces by plotting the differences of **(A–C)** at *t* = 157 ms.

To illustrate the difference in the two decay methods, Figure 4 shows index surface subtracted from the time surface for a single recording from the dataset. The figure shows that the binning time surface has a lower activation than the binning index surface when the speed of the airplane is low (at the start of the recording). As the airplane speeds up through its fall, the total time surface activation continues to increase whilst the index surface remains approximately constant. In fact, at t = 157 ms, the total activation on the time surface is approximately twice that of the index surface which remains relative stable throughout the recording. This stability of index surface activation is the direct result of the decay process. Since both the increase and decrease in surface activation are a function of event index, all decay kernels with a finite impulse response will inevitably generate stable surface activations. This is in contrast to the time decay method where no coupling exists between the activation and decay of the surface. Figures 4D–F show that the difference between the two decay methods are greatest for the binning method, followed by linear decay and finally exponential decay, which is the result of a slight reduction in surface activation from binning to linear to exponential decay for the time surfaces. This reduction is due to the kernel width such that the arrival of new events overwrite the entries for pixels that have recently been activated. This effect is more pronounced for kernels with a longer time window as the surface maintains the value for longer. This same effect is also present in the index surfaces, but is less prominent due to the lower variance of the index-based activation plots. Overall, Figure 4 highlights the event-overwrite effect for different decay methods and kernels, as well as the significantly lower variance of index surface activation in the presence of change in velocity (due to gravity) relative to time surfaces. Such lower variance potentially allows downstream processing stages to be optimized for the stable operating point of the index surface.

### Target Velocity vs. Surface Activation

**Figure 5:**
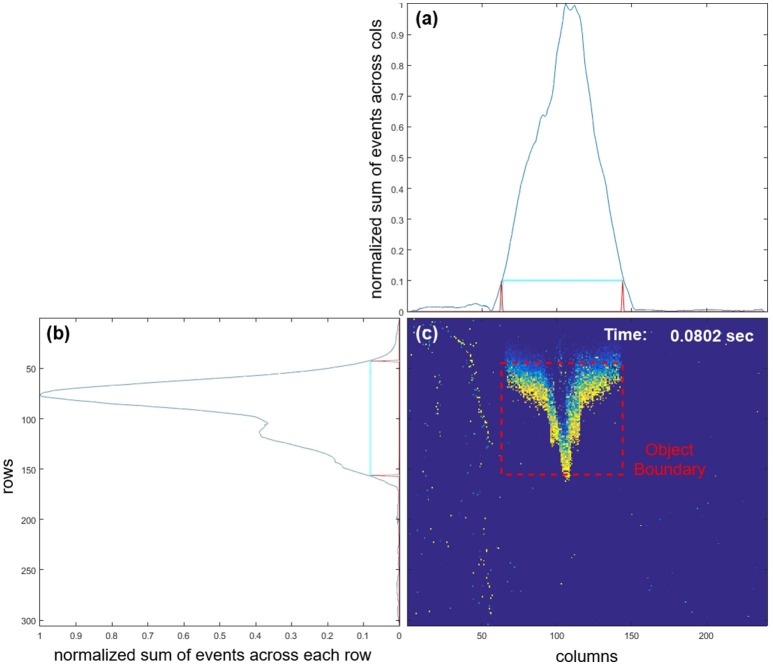
Screenshot from a live demonstration of the airplane drop test after 0.08 s. **(a,b)** are a smoothed summation of recent events across columns and rows, respectively. The smoothing was performed by using an 8-pixel wide rectangular moving average window. Due to the relatively high speed of the airplane these summations, when normalized and thresholded at 0.1, could reliably be used to extract the fast-moving airplane from the static background or slower moving objects. The generated target object's boundary is shown in **(c)**. Note that movement of the body of the author (light vertical trace on the left) as he drops the airplane is slow relative to the airplane and generates relatively few events and so does not reach even the low set (th = 0.1) detection threshold.

Prior to the feature extraction and recognition, the airplane is detected and the location within the field of view is determined. The speed of the airplanes is much faster than any other stimulus expected within the field of view of the camera, such as the body of the author accidently entering the frame, as can be seen in the lower right pane of Figure 5c. Therefore, summation of events across the rows and columns of the camera's field of view (after normalization and thresholding as shown in Figures 5a,b provides a simple method to detect the boundary of the airplane in the limited context of this investigation. While the presence of slow moving objects in the background can be rejected as shown in Figure 5c, complex background objects with similar velocities to the target would impair this simple object detector.

In terms of limitations, the presented dataset is constrained in the sense of having only a single high-speed object in the field of view against an effectively blank background. This restriction allows a more focused investigation of different methodologies as well as of the sources of variance in the data such as target orientation and velocity. While the restriction may appear to limit the generalization of the results to more complex scenes, the dataset and the resulting network solutions should be viewed as investigating a local region within a more complex visual scene and the processing required for it which would be represent a small section in a larger system.

**Figure 6:**
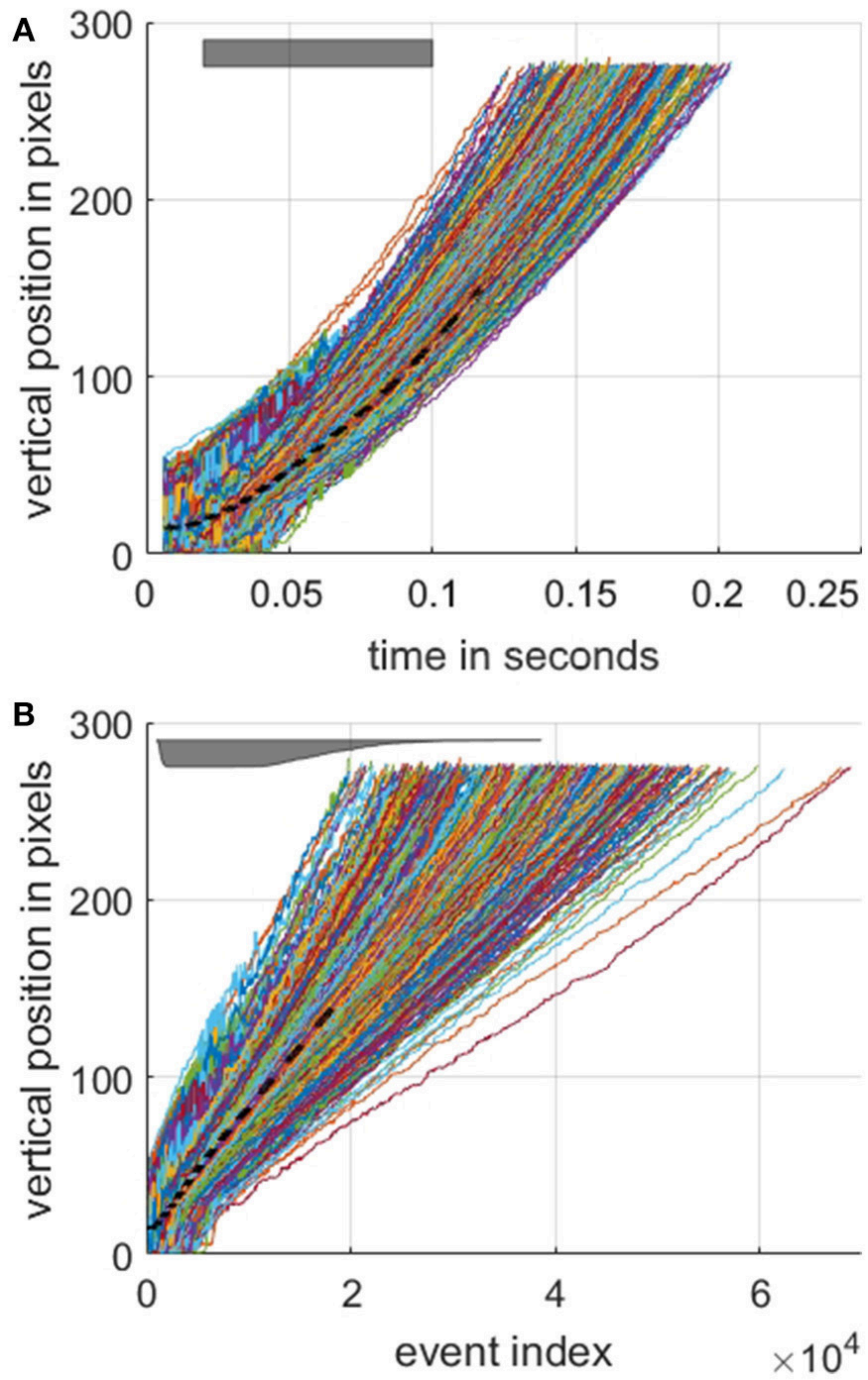
Estimated vertical position of the target as a function of time **(A)** and as a function of event index **(B)**. The dashed black line marks the mean position over all recordings. For the entire dataset, the mean time interval from the first valid object boundary detection event to the last was 156.2 ms with a standard deviation of 17.8 ms. The target's position was defined as the midpoint between the object boundaries as shown in Figure 5 **(C)**. The gray bar at the top left in **(A)** indicates the time window used for investigating the effect of target velocities on surface activation in Figure 7. The same gray time window bar is shown in lower **(B)** panel as a function of event index. The relative thickness of the bar is proportional number of recordings in the time window of **(A)** at each event index. Note the color assigned to each recording profile is arbitrary.

By using the detection method described we can plot the estimated vertical position of each target airplane as shown in Figure 6, both in terms of time in Figure 6A and event index Figure 6B. These vertical position profiles serve to further highlight the difference between the index-based and time-based approaches in the context of local velocity. Whereas, the estimated position plots take on their expected parabolic shape when plotted against time, when plotted against index, the trajectories are linear to a first approximation. The linearity of target position with respect to event index provides an interesting insight into the potential use of index surfaces for tracking, however, this is beyond the scope of the work presented here, which focuses on detection and recognition.

**Figure 7:**
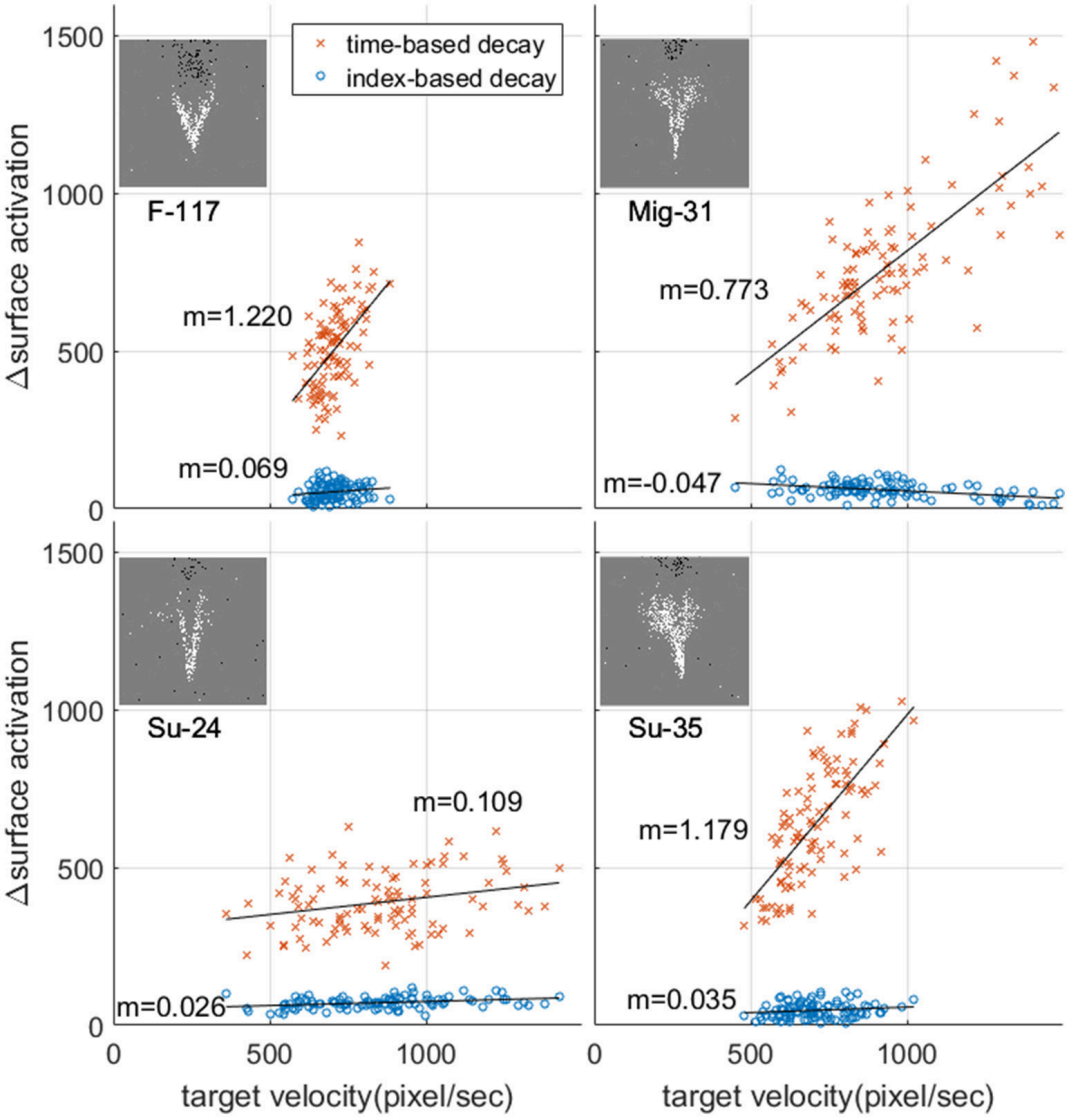
Relationship between change in surface activation and target velocity and the resultant mean rate of change in surface activation. Each point represents a single recording in the dataset. The mean value of target velocity and change in surface activation was calculated over the time window indicated in Figure 6 **(A)**. For each panel m indicates the slope of the line of best fit.

Figure 7 illustrates the wide range of velocities in the dataset and the associated mean rate of change in surface activation for time surfaces, index surfaces. The exponentially decay kernel was used for this test. The line of best fit through the data demonstrates different relationships between velocity and change in surface activation which arise from the different geometries of the airplanes. In all cases, however, surface activation is significantly more sensitive to velocity when using time surfaces than index surfaces. This invariance hints at potential utility of index surfaces for velocity invariant feature generation, where features learnt from a dataset with a particular velocity distribution operate equally well on a dataset with an entirely different velocity distribution, which is not the case for time surfaces. We explore the ramifications of this invariance further in section Velocity Segregated Dataset.

### Event-Based Feature Extraction

An event-based feature extractor was used to learn the most common spatio-temporal features generated by the recordings. The unsupervised spike-based feature extraction algorithm was developed for hardware implementation, as previously described in Afshar et al. (2014). In this algorithm, the Synapto-dendritic Kernel Adaptation Network (SKAN), a single layer of neurons with adaptive synaptic kernels and adaptive thresholds compete in the temporal domain to learn commonly observed spatio-temporal spike patterns. These adaptive synapto-dendritic kernels provide an abstracted representation of the coupling of pre- and post-synaptic neurons via multiple synaptic and dendritic pathways allowing unsupervised learning and inference of precise spike timings. By conceptually combining multiple synapses, the most numerous elements of any neuromorphic system, into a single adaptive kernel, the SKAN algorithm allows an efficient yet reasonably complex model of STDP to be realized in hardware. In Afshar et al. (2015) the algorithm was extended using a simplified model of Spike Timing Dependent Plasticity (STDP) (Markram et al., 1997) to provide synaptic encoding of afferent Signal to Noise Ratio. In Sofatzis et al. (2014) the algorithm was used to perform real-time unsupervised hand gesture recognition using an FPGA. In this work, the event-based approach is continued at the feature extraction layer with the output spike of the winning neuron representing a *feature event*.

The SKAN layer operates via two simple feedback loops: a synaptic kernel adaptation loop and a threshold adaptation loop. Each input event *u*_*i*_*(t)* in a spatio-temporal pattern activates a triangular post synaptic kernel r_*i*_(t) as described by (11) and (12). The kernels are summed at the soma to generate a membrane potential. While this membrane potential is above the neurons adaptive threshold Θ(*t*), the neuron output *s*(*t*) goes high, which is analogous to a series of action potentials or a neuronal burst, as described in (13). While the neuron output *s*(*t*) is high, the kernels perform their temporal adaptation operation as described by (12). According to this rule every time step where the neuron output is high and the kernel is rising (*p*_*i*_ = 1), the synaptic kernel's slope Δ*r*_*i*_ is reduced by a small amount *ddr*, thus moving the kernel peak later in time to better match the observed pattern. Conversely if the event is too early, the kernel's slope Δ*r*_*i*_ is raised contracting the kernel and moving its peak earlier in time.

(11) $$\begin{array}{l} p_{i}\left(t\right)=\{\begin{array}{c} \begin{array}{c} 1\text{ if}\left(u_{i}\left(t\right)=1\wedge p_{i}\left(t-1\right)=0\right)\\ \vee \left(p_{i}\left(t-1\right)=1\wedge r_{i}\left(t-1\right)<w_{i}\right) \end{array}\\ \begin{array}{c} -1\text{ if}\left(p_{i}\left(t-1\right)=1\wedge r_{i}\left(t-1\right)\geq w_{i}\right)\\ \vee \left(p_{i}\left(t-1\right)=-1\wedge r_{i}\left(t-1\right)>0\right) \end{array}\\ \begin{array}{cc} 0 & \text{else} \end{array} \end{array} \end{array}$$

(12) $$\begin{array}{lll} \left[\begin{array}{c} r_{i}\left(t\right)\\ \Delta r_{i}\left(t\right) \end{array}\right] & = & \left[\begin{array}{c} r_{i}\left(t-1\right)\\ \Delta r_{i}\left(t-1\right) \end{array}\right]+p_{i}\left(t-1\right)\left[\begin{array}{c} \Delta r_{i}\left(t-1\right)\\ ddr\times s\left(t-1\right) \end{array}\right] \end{array}$$

(13) $$\begin{array}{lll} s\left(t\right) & = & \{\begin{array}{c} \begin{array}{cc} 1 & \text{if}\sum_{i}r_{i}\left(t\right)>\Theta \left(t-1\right)\\ 0 & \text{else} \end{array} \end{array} \end{array}$$

The neuron's thresholds adapt via a similar mechanism to the kernels. At each time step where the neuron output is high the neuron's threshold also rises. In addition at the falling edge of the neuron output pulse, the threshold falls by a small value. A single inhibitory neuron prevents multiple neurons spiking at the same time thus preventing duplicate learning of the same pattern by multiple neurons.

(14) $$\begin{array}{l} \Theta \left(t\right)=\{\begin{array}{c} \begin{array}{cc} \Theta \left(t-1\right)+\Theta_{rise} & \text{if}\sum_{i}r_{i}\left(t\right)>\Theta \left(t-1\right) \end{array}\\ \begin{array}{c} \Theta \left(t-1\right)-\Theta_{fall}\;\text{if}\sum_{i}r_{i}\left(t\right)\\ \;=0\wedge \sum_{i}r_{i}\left(t-1\right)>0 \end{array}\\ \begin{array}{cc} \Theta \left(t-1\right) & \text{else} \end{array} \end{array} \end{array}$$

This simple hardware implementable rule-set allows the neurons to orient their spatio-temporal receptive fields from a random starting point toward the most commonly observed patterns, thus attempting to optimally represent the observed data given a limited number of features. It is in the class of unsupervised training algorithms used in wide range of neuromorphic algorithms such as STDP. For detailed description of the hardware implementation of the algorithm and resultant behaviors see (Afshar et al., 2014).

**Figure 8:**
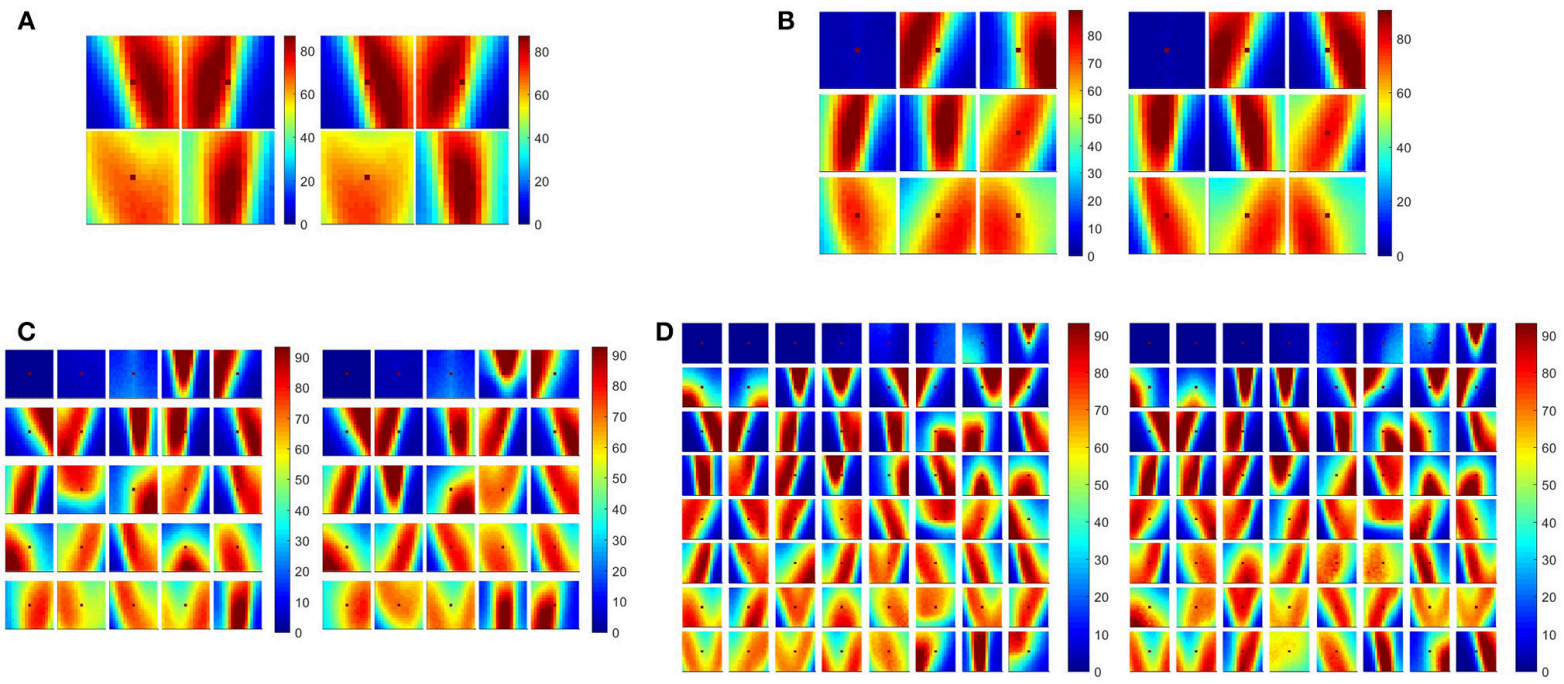
Consistency of feature generation at multiple network scales. **(A–D)** show 4, 9, 25, and 64 spatio-temporal features, respectively, extracted from the ATIS airplane drop dataset. Each panel show results from two independent trials. To allow for visual comparison of the two feature sets, the features from the first trial have been ordered based on the sum of the squares of the weight of each pixel in each feature. The features of the second trial were then sorted based on cosine distance to the first feature set. Only the feature-set obtained from two instances of the time-based, exponentially decaying surface is shown above for brevity. The features resulting from the other kernels resulted in qualitatively similar features dominated by wing edge, nose cone tail features as well as features coding for noise.

When the camera detects a new event, a 13 × 13-pixel region of the surface around it is converted to a temporally coded spatio-temporal spike pattern. This value to time encoding method was originally used in Masquelier and Thorpe (2007). The normalized real-valued intensity of the surface is first rescaled from 0–1 to 0–255 and then mapped to an 8-bit unsigned integer. This 8-bit encoding of the surface allows for potential hardware implementation of the SKAN kernels, without needing floating point operations. This integer representation of the local surface region is then encoded into spike delays forming a spatio-temporal spike pattern. The resultant pattern is then used as the input to a 25-neuron network. The neurons were trained 10 times independently using half the dataset consisting of 50 recordings from each plane type augmented by the left-right flipped version of these recordings. Learning (adaptation) in the feature detection neurons was then disabled. Independent training of SKAN on randomly selected sections of the dataset consistently resulted in similar spatio-temporal features being learnt. The panels in Figure 8 show the resulting feature set from two independent trials at different network sizes to demonstrate this. As the comparison of the trained feature sets shows the same consistent features were learnt at each network size, with the features coding for the leading edge of the airplane nose cones and wings dominating the feature sets. In addition, variants of a solitary noise spike produced often by the ATIS camera are represented as noise features appearing in top left of Figures 8B–D. This consistency was also observed over training epochs of the individual trials. As the number of neurons is increased some of the neurons no longer code for the same features, as can be best seen in the bottom right neurons of Figure 8D. Note also the increasing number of variants of the “noise feature” as the network size is increased. These variants of the “noise feature” encode weak traces of features which are too weak to show in the full color scale.

Of the many network sizes shown in Figure 8 the 25 neuron network was chosen for the investigation of the other parameters in the system. In section Feature Extractor Size and Number, we return to investigate the effect of network and feature sizes in greater detail. Following feature extraction, and with learning disabled, the neurons compete to recognize incoming spatio-temporal event patterns generated from the same 13 × 13-pixel region of the surface following each new event with the spike output of the winning neuron representing a feature event. These feature events were then stored onto 25 separate *feature time surface or feature index surfaces*, which were generated identically to the event surfaces described in section Time-Surface vs. Index Surfaces using the same decay method and decay factor.

### Spatial Pooling of Feature Surfaces

**Figure 9:**
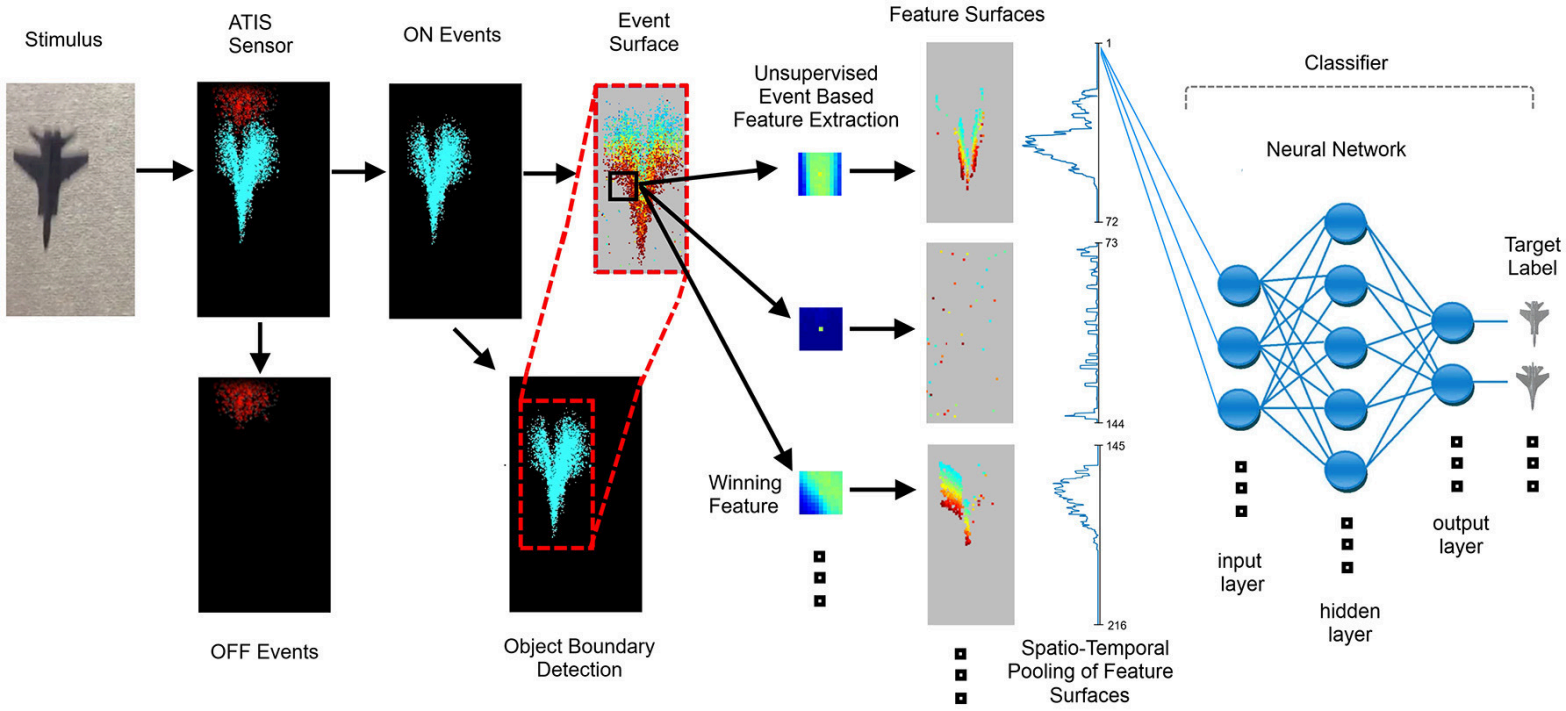
Block diagram of the full event-based detection feature extraction and recognition system. The target is sensed by the sensor and the generated ON events are processed using a time or index surface. Each event triggers a comparison of a local patch around the event with a set of features or neurons. The winning neuron outputs an event which in turn is placed on a feature surface. The feature surfaces are summed across the rows and columns and presented to the back end classifier. The classifier is here depicted as a network with a hidden layer but we also use a linear classifier. Note that in the feature surface pooling stage only the vector summing the feature surfaces across columns is shown, with the second vector showing the summation across rows omitted for clarity.

In order to reduce the required processing and speed up simulation, the subsystems following the feature surfaces were operated in a frame-based manner such that at periodic intervals the estimated target region from each feature surface was sampled to generate feature frames. The interval used for sampling was the same as the time surface decay constant τ_*e*_ = 3 ms. The surface sampling was time-based for both the time and index surfaces so as not to bias the comparison. To reduce the input size to the classifier, spatial pooling of the feature surfaces was performed. To perform this spatial pooling, the estimated object boundary region was summed along the rows and columns, generating two one dimensional feature vectors, one for the rows and one for the columns. The length of these vectors would vary at each feature frame depending on the size of the estimated target region. Thus, in a network with N neurons for each feature a target region of size R rows and C columns would generate two one-dimensional vectors (of length R and C, respectively) resulting from the summation of the image region across rows and columns for each of N surfaces. In order to provide the classifier with a uniform input layer size, the varied length feature vectors R and C need to be resampled to a uniform length. This was done using linear interpolation and the uniform vector length chosen was 72, which, when multiplied by the number of pooling dimensions (2), and the number of features (25), produced a 3,600-input layer for the classifier. The resultant end-to-end system is shown in Figure 9.

### Parameter Selection

In order to fairly evaluate the relative performance in terms of recognition accuracy resulting from different decay kernels, surfaces decay methods, feature extractor numbers, and their receptive field sizes, a large number of free system parameters must first be selected. These parameters, listed in Table 1, are used to implement event and feature surface generation, surface sampling, object detection, feature extraction, spatial pooling, regularization, and classification. In order to ensure that the selected parameters do not advantage the index-surfaces or the feature extraction methods that are the focus of this work, all subsystem parameters would need to be evaluated in terms of their combined effects on the performance of each method under testing. However, this represents a prohibitively large search space to explore in a brute force fashion. Instead, the approach taken in this work to remove possible parameter selection bias in favor of the proposed methods was to optimize all parameters to achieve the highest recognition accuracy on what may be considered the null hypothesis: that simple time-based binning kernels used on raw input events outperform other kernels, decay methods, and feature extractors. To this end, the parameters in Table 1 and all algorithm design choices where selected via a manual heuristic search for optimal recognition performance using the time-based binning surface *BTS*_*i*_ whose spatially pooled output was fed directly to the classifier without the use of feature extractors. The classifiers were then selected for optimal performance on the output data generated by the selected parameters. Once optimized in this way for the “null hypothesis,” these same parameters and network structures were used for all other tests, ensuring that recognition results were biased in favor of the simple time-based binning approach and not those proposed in this work.

**Table 1:** Free parameters used in the system (unless otherwise stated).

**Subsystem**	**Parameter**	**Value**
Surface generation	Time constant	τ_*e*_ = 3 ms
Surface generation	Index constant	*N*_*e*_ = 554 events
Detector	Smoothing window size	8 pixels
Detector	Smoothing window type	Moving average
Detector	Normalized threshold	0.1
SKAN	Number of features	25
SKAN	Number of input channels	13 × 13 = 169
SKAN	Other parameters	Same as Afshar et al. (2014)
Classifier	Input size using raw event surface (E)	72 × 2 = 144
Classifier	Input size feature event surfaces (F)	72 × 25 × 2 = 3,600
Classifier	ELM hidden layer size	30,000 Neurons
Classifier	Surface sampling interval	3 ms

### Classification

#### Choosing Classifiers

The choice of a back-end classifier used to map feature outputs to classes can play a critical role in the performance of a convolutional feature extraction layer or network. Well-regularized high capacity classifiers with internal non-linearities can provide significant improvement in performance over and above the underlying feature extractors used. In many proposed event-based recognition systems, only a single type of classifier is tested and often only a single instance of such a classifier (the best performing configuration) is reported. While this approach encourages greater attention to the presented work, it can also overstate the performance of the overall system, due to fine tuning. What's more, the use of well-optimized powerful classifiers without concurrently testing simple linear classifiers obscures the role of the event-based feature extractors in the system performance. Here, we propose a dual classifier testing protocol, which ideally should be applied before and after each stage of processing, to provide insights into the effectiveness of the elements under test. For the baseline test, a simple linear classifier is used to measure how linearly separable the underlying data is before and after processing. In addition to this baseline classifier we utilize a large capacity ELM, which, by virtue of the large number of random hidden layer neurons, is likely to project the non-linearities of the dataset into a linearly separable higher dimensional feature space. In addition, the lack of feature learning in the ELM allows a reasonable unbiased estimate of the residual non-linearity in data. This framework of testing provides significant insights, as detailed in the results section, which would not be revealed if only the results from the best performing classifier were reported.

To evaluate the performance of the system, two measures of recognition accuracy were considered: per-frame accuracy and per-drop accuracy. For the per-frame measure, the feature vectors described Section Event-Based Feature Extraction were presented to the classifier at periodic time intervals τ_*e*_. At each frame, the class with the largest output was selected as the winner for that frame. For the per-drop accuracy measure, the class with the highest number of per-frame during the entire recording was selected.

**Figure 10:**
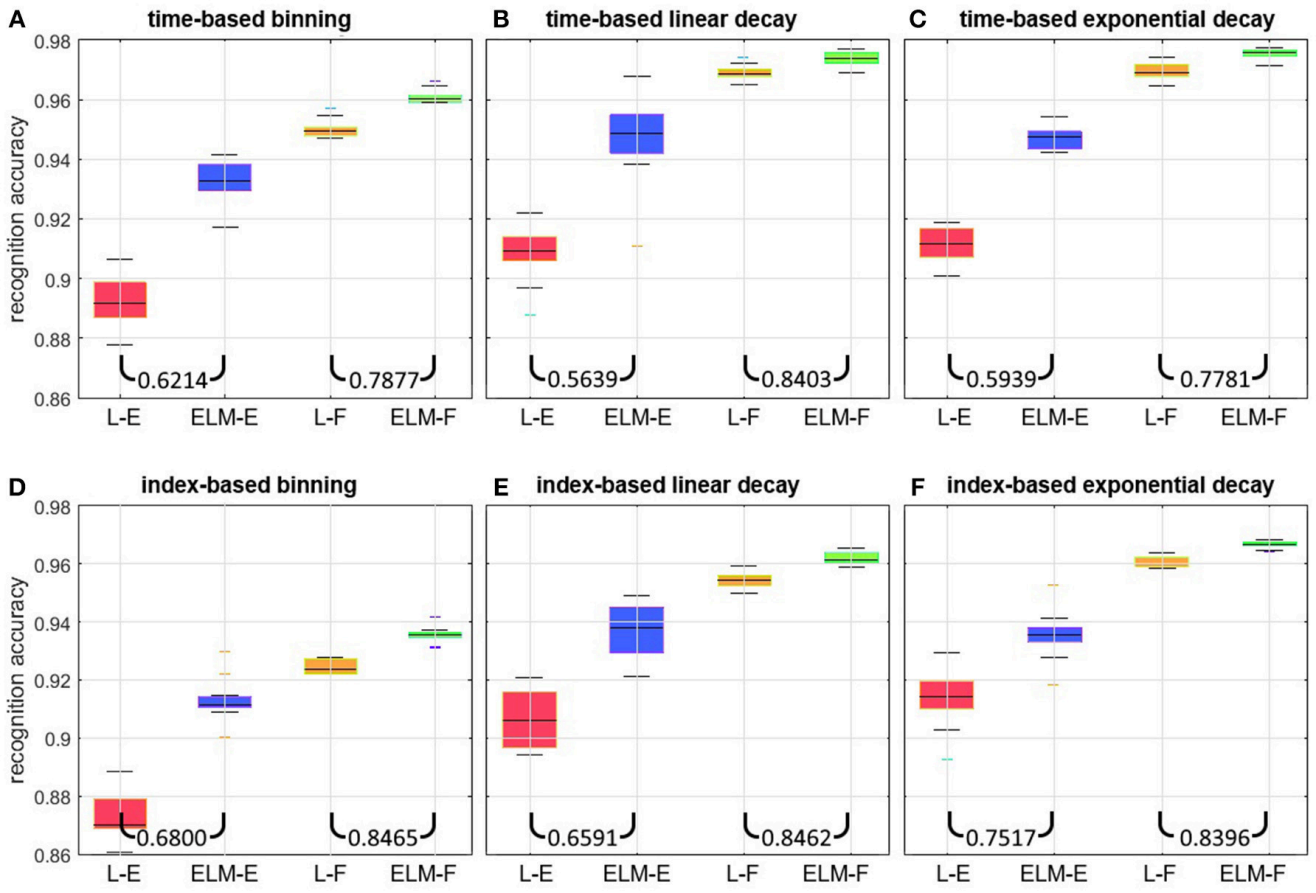
Per-frame recognition accuracy on the full dataset over *n* = 20 independent trials. Each panel shows results from four network arrangements. In (L-E), and (ELM-E) the linear classifier and the 30 K hidden layer ELM chosen in section Choosing classifiers operate on inputs from raw event surfaces. In (L-F), and (ELM-F) the same classifiers use 25 feature surfaces as inputs. Each panel shows results for a different surface generation method: The top three panels show time-based methods using **(A)** binning, **(B)** linear decaying, and **(C)** exponentially decaying surfaces. The bottom three panels show corresponding index-based binning **(D)**, linear decaying **(E)**, and exponentially decaying surfaces **(F)**. The two ratios at the bottom of each panel indicate the median error ratio of the ELM over the linear classifier.

A linear classifier and an Extreme Learning Machine (ELM) classifier (Cohen et al., 2017) with a hidden layer size of 30,000 neurons were trained using the time-based binning method to achieve the highest per-frame recognition accuracy. Figure 10 details the results from this parameter search and the selected classifiers.

## Results

### Results on the Full Dataset

The per-frame recognition results on the full dataset are shown in Figure 10. For each of the panels, the same performance pattern is observed: when operating on raw event surfaces as inputs, the large capacity ELM (ELM-E) significantly outperforms the linear classifier (L-E). This demonstrates the non-linearity of the classification boundaries in this case. In comparison, when feature surfaces are used as inputs, the improvement margin gained by the ELM (ELM-F) is small relative to the linear classifier (L-F) suggesting that the output of the 25 feature extractors is significantly more linearly separable, with less room for improvement through further non-linear expansion. Also noteworthy is that the linear classifier operating on feature surfaces (L-F) outperforms the ELM operating on the event surfaces (ELM-E) for all surfaces generation methods. This shows that the application of a small number of trained local feature extractors is more effective than using a much larger globally connected network of neurons with random input weights. The ratio of errors between the ELM and the linear classifier indicated at the bottom of each panel quantifies this reduction in error for each case.

Comparing the results across the panels for the linear classifier operating on events (L-E), the exponentially decaying surfaces outperform linear surfaces by a margin of 1.75% for the index surfaces and 0.24% for the time-surfaces. In turn the linear surfaces outperform the binning method by 3.06 and 1.36% for the index surfaces and time surfaces, respectively. For the case of the linear classifier operating on feature surfaces (L-F), the exponentially decaying surfaces outperform linear surfaces by a margin of 0.57% for the index surfaces and 0.22% for the time-surfaces, and in turn the linear surfaces outperform the binning method by 3.07 and 1.91% for the index surfaces and time surfaces, respectively. Also, consistently, the improvement of exponential kernels over linear kernels is not as significant as their margin with the binning method.

It is worth noting that, when the ELM is chosen as the back-end classifier, the margin in performance improvement obtained from feature extraction is reduced. This is to be expected, since the randomly situated hidden layer neurons of the ELM have a greater chance of improving the linear separability of segments of the dataset, if such segments are not already linearly separable due to processing in the preceding layer. This effect of obscuring the performance of other subsystems is not limited to the ELM. A similar effect would be expected with any other classifier performing non-linear expansion. This underlines the need to include results from a simple linear classifier when comparing alternative systems. Also worth noting is that for the preceding results (features outperforming raw events, and exponential and linear kernels outperforming binning) all system parameters were optimized for the time-based binning method. These results therefore confirm the suitability of exponential kernels for time and index-surface generation. This conclusion is also supported by results in Akolkar et al. (2015), where the information from the visual scene is found to rapidly rise within a small initial temporal window, but thereafter fall gradually with increasing window size, as is best described by an exponentially decaying kernel. By weighing events in an approximately compensatory manner to their information content as described in Akolkar et al. (2015), the exponentially decaying kernel results in the highest information content for the classifier. Another observation from Figure 10 is that all time-based decay methods outperform the index-based decay methods by ~1% on the full dataset with the largest performance disparity observed between the index-based binning method *BIS*_*i*_ and the time-based binning method *BTS*_*i*_. This would be expected, since the later method was used during all parameter optimizations and would be most advantaged by the selected parameters. Based on the results shown in Figure 10 we narrow further investigations by selecting linear classifiers L-E and L-F and focus on exponentially decaying surfaces *EIS*_*i*_ and *ETS*_*i*_.

### Frame Balanced Dataset

**Figure 11:**
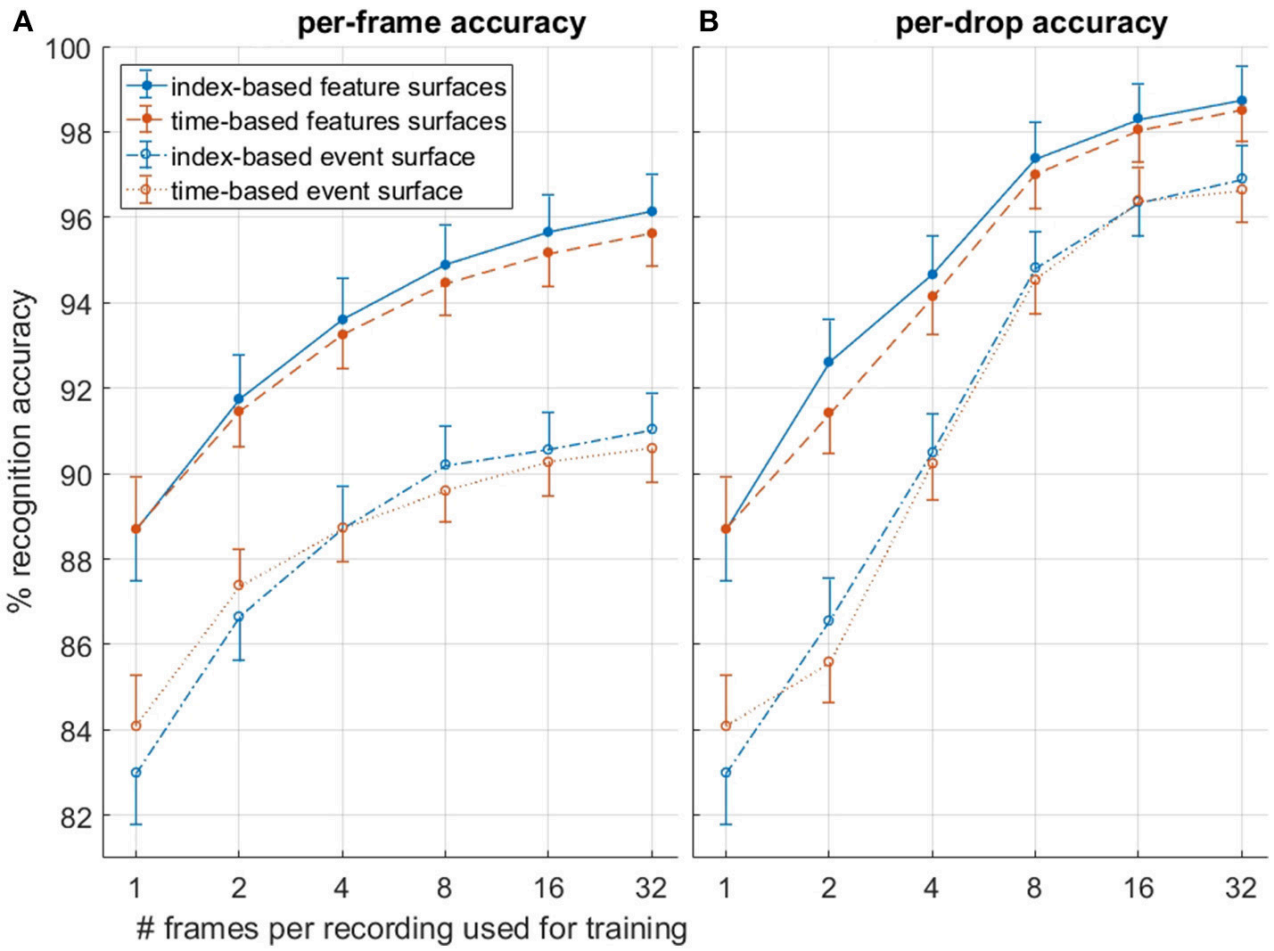
Comparison of **(B)** per-drop and **(A)** per-frame recognition accuracy as a function of the number of randomly selected frames used during training from each recording. The index-based *EIS*_*i*_ surface and time-based *ETS*_*i*_ surfaces are compared. Results shown are over *N* = 20 trials. A linear classifier was used in all test.

In order to generate a balanced dataset, an equal number of frames from each recording was selected. In this way, the total number of presentations to the classifier for each class was equalized. As Figure 11 shows 1, 2, 4, 8, 16, and 32 frames were sampled from each of the airplane recordings and presented to the linear classifier operating on events surfaces L-E and feature surfaces L-F for each of the *EIS*_*i*_ and *ETS*_*i*_ surfaces.

**Figure 12:**
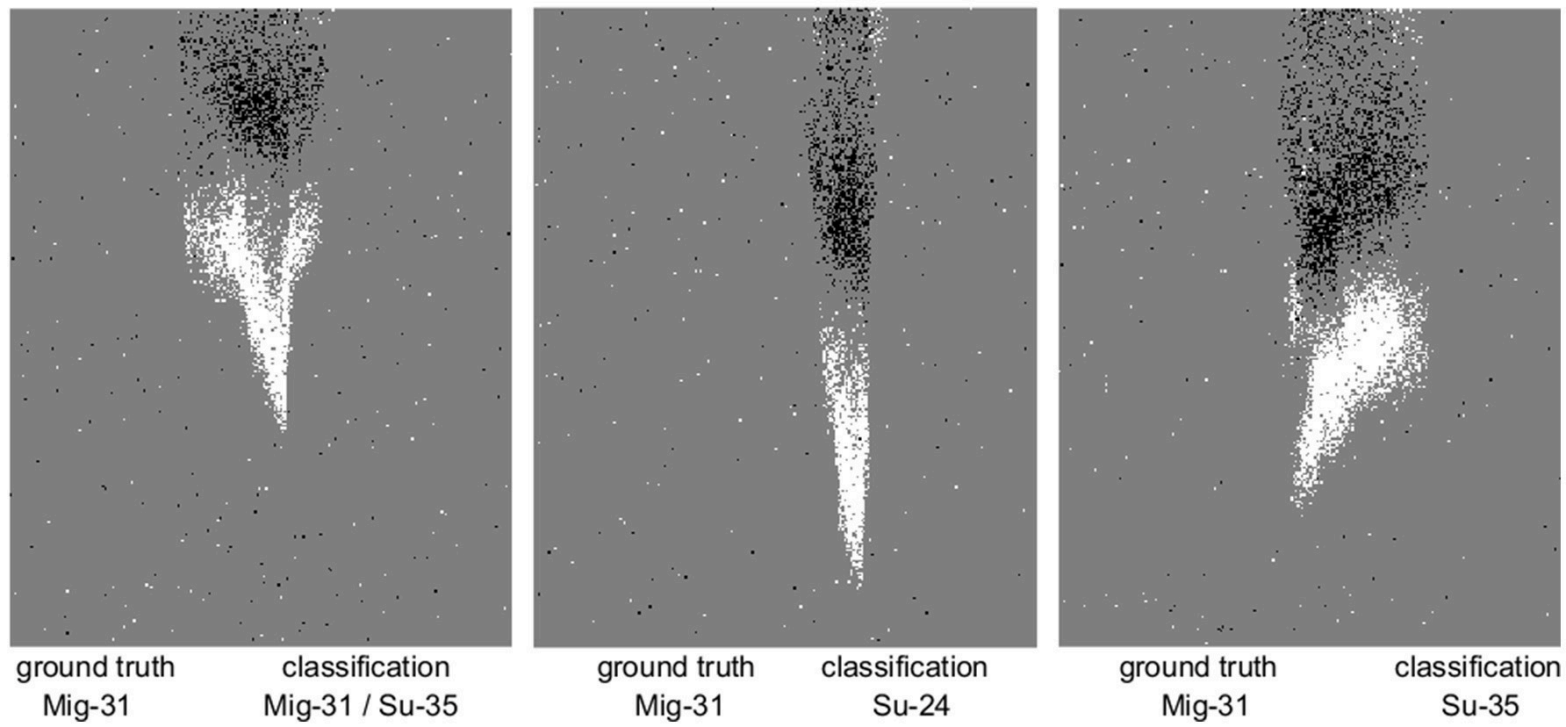
The three drops misclassified by an instance of a linear classifier using 25 exponentially decaying index-based feature surfaces. Captured frames show airplanes at mid-point (in time) of recording.

As Figure 11 shows, both the per-frame and per-drop accuracy increase as a function of the number of frames used during training. Additionally a sharper increase and higher final accuracy is observed for the per-drop accuracy measure, as would be expected, since the per-drop measure is analogous to a max pooling operation which benefits from increased pool size. The relative performance margin of the network using feature surfaces over raw event surfaces is reduced in the per-drop measure, as more information is accumulated over a recording, reducing error, and approaching the 100% accuracy upper bound. The highest number of random frames used per recording was 32, as this was approximately equal to the total number of frames in the shortest recording (see Figure 2B). Table 2 details the accuracy results for this balanced dataset while Figure 12 shows misclassified recordings for one instance of the highest performing network using index-based decaying feature surfaces and a linear classifier, illustrating that some drops are almost impossible to classify correctly.

**Table 2:** Per-frame and Per-drop accuracy results on the frame balanced dataset for four selected systems: Linear classifier operating on events surfaces (L-E) and feature surfaces (L-F) for each of the **EIS**_**i**_ and **ETS**_**i**_ surfaces.

	**Per-frame (%)**	**Per-drop (%)**
Time-based Event surface	90.60 +/−1.02	96.64 +/−1.47
Index-based Event surface	91.03 +/−0.89	96.90 +/−1.34
Time-based Feature surfaces	95.64 +/−0.79	98.52 +/−0.75
Index-based feature surfaces	96.15 +/−0.84	98.75 +/−0.78

*Number of trials used is 20*.

Interestingly, in contrast to the full unbalanced dataset results detailed in section Results on the Full Dataset, the per frame balanced results in Figure 11 and Table 2 show little significant difference in accuracy between the index-based and time-based surfaces for either the per-frame or per-drop measures, suggesting that the observed slight advantages in accuracy on the full dataset may be due to the use of time-based surfaces during parameter selection of section Parameter Selection and linked to imbalances in the number of frames per recording present in the full dataset for the two different methods.

### Velocity Segregated Dataset

**Figure 13:**
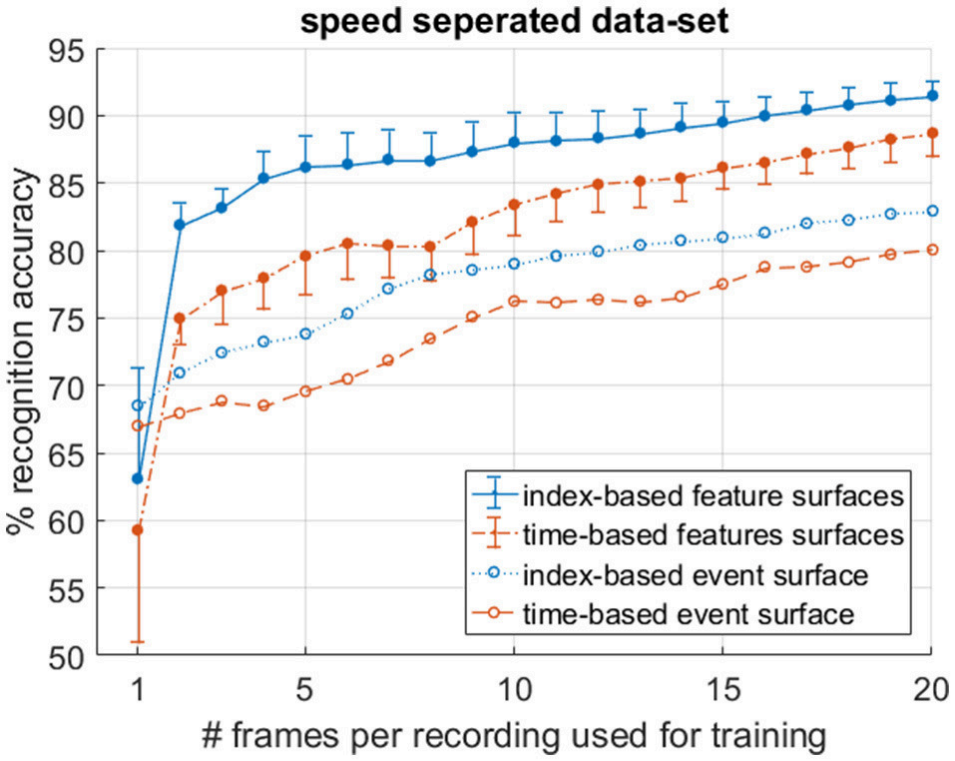
Mean and standard deviation per-frame accuracy on a speed segregated dataset over 10 trials clearly demonstrates superior performance of index-based surfaces in the presence of velocity varying data.

As outlined in section Target Velocity vs. Surface Activation, the apparent velocity invariance property of index surfaces motivates a test using a modified dataset which is split in terms of target velocity. Thus, in order to compare index-based and time-based surfaces in terms of target velocity invariance, the recordings were divided into 200 “slow” and 200 “fast” recordings based on the estimated vertical airplane velocity at the midpoint (in time) of each recording. Since the airplanes speed up during the fall, the system was trained on the n-first (slowest) frames of the slow recordings and tested on the n-last (fastest) frames of the fast recordings. In this way, by varying the number of frames n, datasets with different degrees of velocity segregation could be tested. The resulting recognition accuracies in Figure 13 demonstrate that with increasing n, and thus decreasing velocity segregation in the data, the recognition accuracy of all systems rise. Figure 13 further shows that although training on a speed segregated dataset significantly reduces accuracies for all systems in comparison to training using a randomly sampled dataset (such as shown in Figure 11), the decline is significantly larger for time-based decaying surfaces. This difference demonstrates the relative robustness of index-based decay surfaces to variance in velocity and their utility in applications where the full range of potential target velocities to be encountered during testing is not available in the training data.

Therefore, given the results in the previous section, it can be concluded that, at the local scale, with a single target in the field of view, systems using index-based decay surfaces tend to match equivalent systems using time-based decay surfaces, when presented with an adequately wide range of velocities in the training data, since their advantage of velocity invariance is effectively neutralized. But when the available range of velocity distributions for training is incomplete, index-based decay surfaces tend to produce more robust performance. Given this finding, and in order to limit the scope in the next section, we narrow our focus exclusively on index-based surfaces and investigate the effect of different feature extraction networks and their effect on recognition accuracy. This is also supported by findings in Ghosh et al. (2014), where a small superiority was found when using fixed event windows over time windows. However, those tests were performed using a randomly sampled training set, likely containing data with velocity distributions that were similar to the test set. As such their results are similar to the full dataset results examined in section Frame Balanced Dataset of this work, which only showed a slight improvement due to the velocity variance available in the training dataset. In this work, by additionally testing the algorithms using a range of velocity segregated datasets, the robustness of the index surface method is more completely investigated.

### The Decay Constants

**Figure 14:**
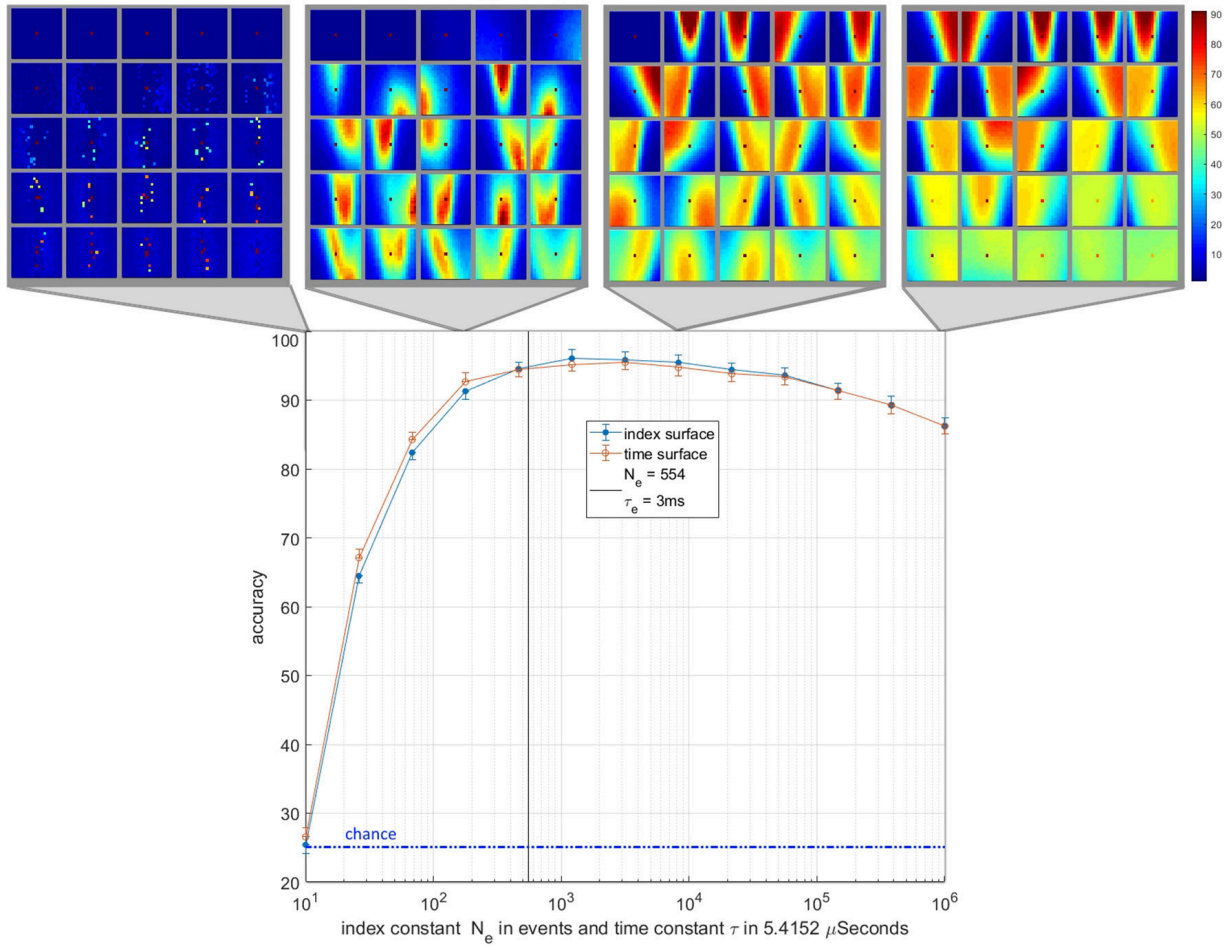
Classification accuracy and typical feature sets as a function of the decay constants for time and index surfaces. The lower panel shows accuracy plotted against the index decay constant *N*_*e*_on a logarithmic scale. The time surface results are plotted on the same logarithmic scale where a 1event to 5.4152 μs conversion rate is used to align the results. This conversion rate is based on the average event rate over the entire dataset. The vertical solid line at *N*_*e*_ = 554 and τ_*e*_ = 3 ms (τ_*e*_ = 554 × 5.4152 μs) indicates the value of the index and time decay constants used in rest of the work. The horizontal dotted line indicates chance accuracy. All tests were performed over *N* = 20 independent feature extraction trials. The feature sets above the panel show instances of the feature sets for four points on the decay constant axis. The feature set shown are from index-based surfaces.

An important element of any event-based surface is the value of its decay constant. In this work the value of decay constants, τ_*e*_ = 3 ms and *N*_*e*_ = 554 events were effectively chosen arbitrarily. This raises an important question about the optimality of the chosen decay constants and the robustness of the generated features and recognition accuracy to different values of these decay constants. A closely related question, which applies only to index surfaces, is whether targets which generate more or fewer events, e.g., due to different object size or contrast, could still be learnt and recognized with the decay constants chosen. To investigate these questions a wide range of decay constants across six orders of magnitudes were tested on a frame balanced randomized training and testing dataset. The resulting recognition accuracies and selected feature sets are shown in Figure 14. The results show a similar pattern for time and index surfaces with little significant difference in accuracy. At the extreme decay rate of 10 events and 54 μs the systems perform little better than chance, since virtually all event information is decayed away before it can be extracted. This leaves all the features coding for variants of the noise feature. As the decay constant increases to by two orders of magnitude, coherent features begin to emerge coinciding with a rapid increase in recognition accuracy. At this event rate there are still multiple features coding for a single noise spike. Index decay constants of between three and four orders of magnitude of events correspond with a plateau in recognition performance. This region coincides with the range where the noise feature is only represented by one or two neurons with all remaining neurons coding for complex features. After four orders of magnitude increase in the decay constant, the accuracy begins to decline slightly. In this region the noise features begin to be represented once more but this time with a highly activated background which is a direct result of the much slower decay rate.

As Figure 14 illustrates, when sweeping the decay constant, the number of variants of the noise feature in the network roughly correlates to the feature extraction performance of the network. The feature set with the fewest representations of the noise feature (ideally only one) performs the best. This is expected since the noise feature is unlikely to be correlated to any particular class of object and the frequency of its representation in a feature-set reduces the efficiency of that feature-set, leaving fewer neurons to represent classification relevant feature information. Figure 14 also shows a wide central region of stable performance that is robust to the choice of τ_*e*_ and *N*_*e*_. The results also show that over estimating the optimal value of the decay constant is less harmful than under estimating with significantly less reduction in accuracy.

### Feature Extractor Size and Number

**Figure 15:**
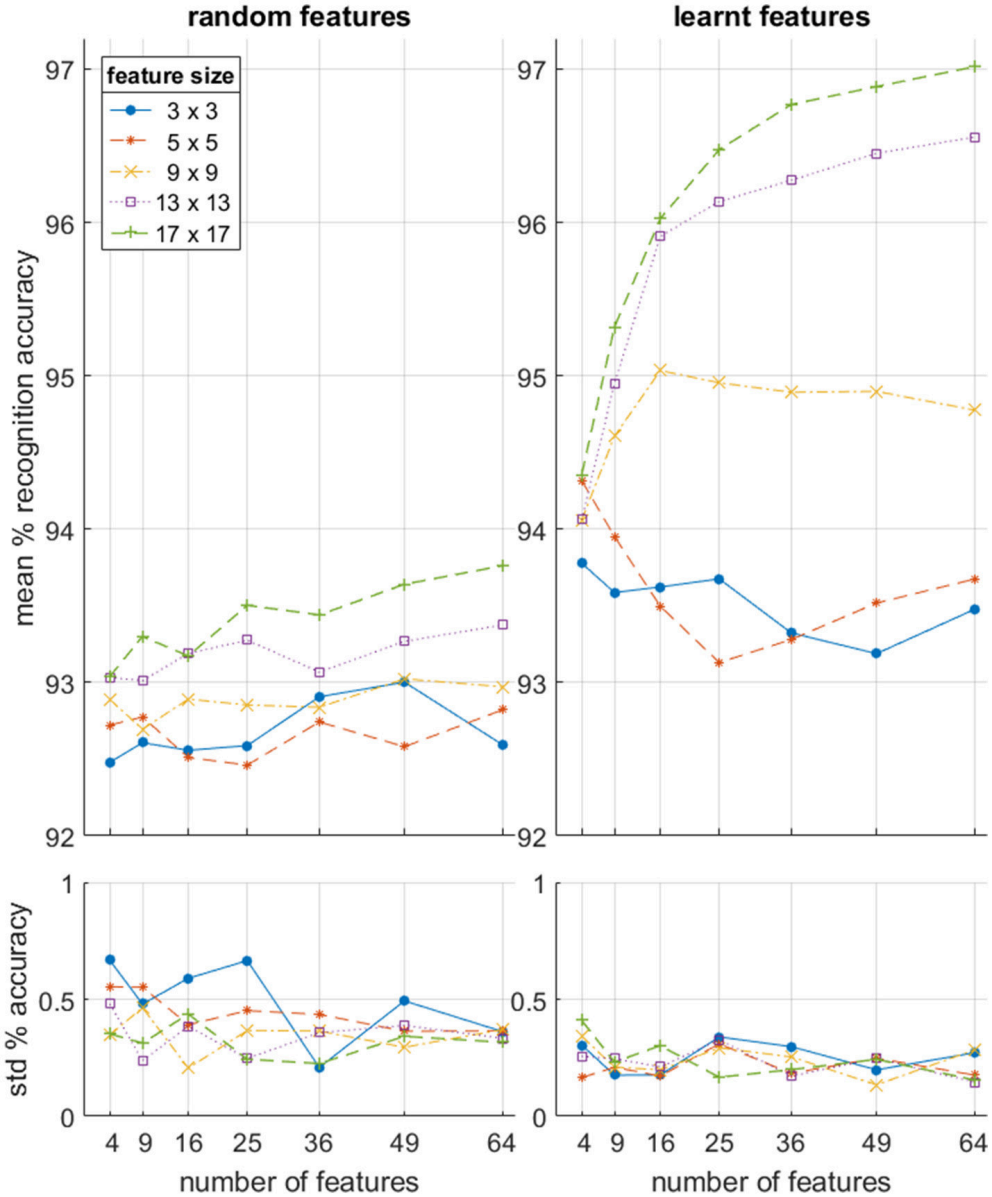
Per-frame accuracy on the full dataset as a function of feature size and number of features used in the feature extraction layer for both learnt and random features. *N* = 10 independent feature sets with 10 cross validating classifications per feature set. Note that the baseline linear accuracy using the raw event surface with no feature extraction layer was 91.38 +/− 0.81% as shown in Table 2.

In order to characterize the effectiveness of the feature extraction subsystem in an unbiased manner, a range of feature sizes and a number of feature extractors were investigated and assessed in terms of the resultant recognition accuracy. In addition, for each point on the feature size-feature number space, the results of the learning algorithm described in section Event-Based Feature Extraction was compared to those of equivalent sized networks using random feature sets. The mean accuracy results in Figure 15 (top panels) demonstrate that learnt features outperform random features at every scale while exhibiting slightly lower variance in accuracy (bottom panels).

In addition, while the results from the random features suggest a slight trend toward increased accuracy as a function of both feature numbers and feature size, the learnt feature results clearly show that the larger feature sizes (17 × 17 and 13 × 13) generate higher accuracy with increasing number of features, while the smallest feature sizes (3 × 3 and 5 × 5) exhibits a weak downward trend with the number of features. When the feature size is small, only a few distinct combinations exist. Therefore, when and a large number of them are trained, several features will be very similar, resulting in near identical input generating very different input to the classifier. This reduction in accuracy resulting from the addition of more redundant features is due to the OR operation which must be performed by the back-end classifier. This insight demonstrates that convolutional features layers can, if poorly configured, “over-fit” the data by representing overly specific variants of the same pattern. This effect only becomes apparent with the combined use of a large number of feature, small feature sizes, and relatively small datasets. But this might be an issue in future applications of event-based convolutional networks, where resource efficiency of a hardware implementation may allow a very large number of features in a layer to be trained (especially in the first layer) while the level of independent features in the recorded data may be limited.

We can also note that for both the random and learnt feature sets, the feature size has little effect on accuracy when the number of features becomes very small. This is because there is very little additional discriminatory information that can be captured by the larger sized features when a wide range of unrelated, heterogeneous spatio-temporal patterns become effectively averaged together to generate the (too) few features used in the network. Thus, local spatial complexity of observed data determines optimal feature size and feature number relationships, which, if not considered during hardware implementation, can result in inappropriately scaled network architectures and effectively wasting hardware resources.

## Discussion

While binning methods examined in this work were shown to perform less well than linearly decaying surfaces and exponentially decaying surfaces, the significantly simpler implementation of the binning method allows for much more efficient implementations of event surfaces in neuromorphic hardware. In a similar fashion, the selection of feature sizes and number of features implemented at any layer of a multi-layer event-based network generates trade-offs between hardware resource and performance. In this context, the network and feature size investigations presented here provide guidelines for such network designs.

The four class dataset presented allows reasonably accurate classification using a single layer of feature extraction in combination with a linear classifier; the task can be made increasing difficult by increasing the number of classes in the dataset. In such a case the output of the feature extraction layer would retain significantly greater residual non-linearity. This would increase the performance of gap between the linear classifier and the large ELM. Conversely adding additional feature extraction layers will work in the opposite direction, producing output that is more and more linearly separable and thus reducing the performance gap between the linear classifier and ELM.

The presented recordings in the dataset were varied to cover a wide range of target speeds. As a result any random splitting of training and testing data provided an overlapping range of target speeds in both set. This overlap removed any advantage of index-based decaying surfaces which provide robustness to target velocity. However, in many applications, such as the SSA applications of Cohen et al. (2017), the range of velocities in the training set is limited so that features trained on this limited set of target velocities must generalized to a wide range of as yet unobserved velocity profiles. In this work, such a condition was simulated by iteratively segregating the data based on speed to highlight the utility of the index-based decay method.

One weakness of the index-based decaying method is that it can only be used locally (or globally but on a single target). If events from other non-target object cause a decay in the surface activation of the target, vital information may be lost. Such information loss is not present if target segregation has already occurred via an upstream system, or, more generally, if the surface decay mechanism is viewed as a local mechanism acting on a sub-region of a larger global surface. As such, the presented dataset and the resulting performance of the index-based systems can best be viewed as focusing on a locally operating subsystem within a larger processing system. When viewed as a rigorous analysis of such a central building block of a larger event-based network the value of the investigation presented here becomes more apparent. On the other hand, if a system needs to operate with a single decay method, then the standard time-based decay mechanism would be more optimal, as it can process the entire surface in a global manner.

## Conclusion

In this work, we investigated in detail an event-based feature extraction layer. In order to rigorously investigate the effects of different kernels, decaying methods, classifiers, and feature sizes and numbers, we limited the exploration to a single layer network. Yet the design of deeper networks can be informed by these single layer results. Using a dataset featuring a range of target shapes, scales, orientations, and velocities, it was observed that exponentially decaying kernels outperform other kernels, and that index-based decaying surfaces perform equally as well as time-based decaying surfaces, when robustness to target speed is not required, and outperform them when it is required. We also showed a clear superiority of learnt features over random features and showed that the largest networks of neurons with the largest receptive fields using the most complex kernels outperform all other configurations.

## Author Contributions

SA, GC, and TH designed dataset. SA and GC generated the dataset. SA and GC performed pre-processing. SA, GC, JT, and AvS designed the algorithms. SA implemented the algorithms. SA analyzed the data and results. SA wrote the manuscript. All authors assisted in editing.

### Conflict of Interest Statement

The authors declare that the research was conducted in the absence of any commercial or financial relationships that could be construed as a potential conflict of interest.
